# LRRK2-mediated NLRC4 phosphorylation differentially regulates IL-1β/IL-18 secretion

**DOI:** 10.3389/fimmu.2025.1675137

**Published:** 2025-10-30

**Authors:** Sharmina Deloer, Ivan Fuss, Portia Gough, Anketse Debebe, Mellissa Picker, Robert J. Devita, Inga Peter, Warren Strober

**Affiliations:** ^1^ Mucosal Immunology Section, Laboratory of Clinical Immunology and Microbiology, National Institute of Allergy and Infectious Diseases (NIAID), NIH, Bethesda, MD, United States; ^2^ Host Microbe Symbiosis Unit, Laboratory of Clinical Immunology and Microbiology, Division of Intramural Research, National Institute of Allergy and Infectious Diseases (NIAID), NIH, Bethesda, MD, United States; ^3^ Department of Genetics and Genomic Sciences, Icahn School of Medicine at Mount Sinai, New York, NY, United States; ^4^ Department of Pharmacological Sciences, Drug Discovery Institute, Icahn School of Medicine at Mount Sinai, New York, NY, United States

**Keywords:** NLRC4, LRRK2, inflammasome, Crohn’s disease, inflammation

## Abstract

In the present study, we explored the relation of LRRK2-kinase phosphorylation of the NLRC4 inflammasome to NLRC4 inflammasome function in normal humans and mice, as well as in patients with Crohn’s disease (CD). We found that LRRK2-kinase was both necessary and sufficient for NLRC4 phosphorylation in human mononuclear cells and likely in murine mononuclear cells as well. In addition, such phosphorylation requires ASC association with the nascent NLRC4 inflammasome and is necessary for ASC function. Finally, we found that inhibition of LRRK2-kinase phosphorylation of NLRC4 impairs inflammasome IL-1β production but has little to no effect on its IL-18 production. The mechanism of this dichotomy was revealed in studies of NLRC4 inflammasome activity, showing that pro-IL-1β cleavage is partially dependent on LRRK2-mediated ASC binding and cleavage function, whereas pro-IL-18 is independent of such ASC function. In accompanying studies of circulating cells from patients with CD, a disease associated with LRRK2 polymorphisms that affect LRRK2 expression, we showed that patient cells exhibited increased NLRC4 inflammasome activation; in addition, inhibition of LRRK2-kinase impaired IL-1β secretion but had little or no effect on IL-18 secretion by patient cells. Finally, studies of WT mice or mice with epithelial cell-specific NLRC4 deletion revealed that NLRC4 inflammasome activation causes impairment of gut barrier function that is abrogated by inhibition of LRRK2-kinase activity. Thus, NLRC4 inflammasome function is increased in CD, and its regulation by an LRRK2-kinase inhibitor is calibrated to prevent NLRC4-mediated barrier dysfunction.

## Introduction

LRRK2 (leucine-rich repeat kinase-2) is a multi-domain protein that is encoded by a gene subject to polymorphisms and/or mutations linked to IBD, Parkinson’s disease, and leprosy (1, 2). These genetic associations are likely to be due, at least in part, to the influence that LRRK2 has on immune responses and inflammation via its kinase function. LRRK2, for instance, inhibits autophagy by facilitating Beclin-1 phosphorylation and thereby exerts a positive effect on TLR and NLR-induced NF-κB activation (3, 4). In addition, despite earlier studies to the contrary, it is now clear that LRRK2 kinase activity leads to phosphorylation of NFATc2 and thus promotes its nuclear translocation and cytokine transactivation (5, 6). Yet other pro-inflammatory (kinase-related) functions of LRRK2 include its enhancement of NOD1/NOD2 signaling via up-regulation of RIPK2 phosphorylation (7) and, most relevant to the present study, its optimization of inflammasome activation. The latter, resulting in increased IL-1β production, occurs either via its autophagy-related effect on the NLRP3 inflammasome (8), or on its effect on the NLRC4 inflammasome via NLRC4 phosphorylation (9).

The involvement of LRRK2 kinase function in inflammasome activation may provide insight into its role as a driver of gut inflammation in IBD and, by extension, Parkinson’s disease. The latter is the case because gut inflammation is thought to be a breeding ground for cells that migrate into the brain and abet neuronal loss at this site (10). With respect to LRRK2 enhancement of NLRP3 inflammasome activation (via effects on autophagy) a mixed picture emerges; on the one hand, deletion of this inflammasome in mice most often results in protection from experimental gut inflammation probably due to loss of anti-inflammatory mechanisms such as inflammasome support of epithelial barrier function or Treg generation (11, 12); on the other hand, enhancement of NLRP3 inflammasome activation due to various gene mutations is sometimes (but not always) associated with IBD-like disease (11, 13). With respect to LRRK2 support of NLRC4 inflammasome activation, it is not yet clear that this inflammasome plays an important role in IBD inflammation, since it is commonly thought that it is activated by pathogenic flagellated organisms that are not involved in IBD pathogenesis. However, contrary to this concern, there are recent reports that organisms capable of NLRC4 inflammasome activation and that are capable of enhancing experimental gut inflammation do, in fact, occur in the IBD-associated gut microflora (14, 15). Perhaps more importantly, the NLRC4 inflammasome can be activated by substances released by dying cells, such as lysophosphatidylcholine (LPC), and thus activation of this inflammasome is an inevitable result of inflammatory tissue destruction that is not dependent on infection by certain organisms (16, 17).

The enhancing effect of LRRK2 on NLRC4 inflammasome activation mentioned above is itself not fully understood since there is some controversy regarding the importance or need of NLRC4 phosphorylation in NLRC4 inflammasome activation and function. Thus, whereas Qu et al. (18) found that macrophages bearing an NLRC4 mutation (S533A) that precluded its phosphorylation (at Ser533) were unable to assemble the NLRC4 inflammasome in response to *S. typhimurium* exposure, Tenthorey et al. (19), found that macrophages expressing NLRC4 with the same mutation displayed little or no loss of inflammasome function as evaluated by macrophage pyroptosis or that mice bearing this mutation showed no loss of *in vivo* resistance to *S. typhimurium* infection. Studies by Liu et al, referred to above and showing that LRRK2 phosphorylates NLRC4 at Ser533 also bear on the role of NLRC4 phosphorylation in NLRC4 inflammasome activation since these authors quite clearly found with *in vitro* studies that such phosphorylation augments NLRC4 inflammasome IL-1β generation (9).

In the present study, we explore the relation of LRRK2 kinase-mediated NLRC4 phosphorylation to NLRC4 inflammasome activation anew. We found that phosphorylation does indeed optimize such activation, but this applies to IL-1β production and has little to no effect on IL-18 secretion. Analysis of the mechanism of this differential effect disclosed that phosphorylation affects ASC function and whereas IL-1β production is partially dependent on such function, IL-18 production is not. Importantly, this dichotomy was found to hold in studies of cells from patients with Crohn’s disease, one of the two major forms of IBD. Finally, we found that systemic NLRC4 inflammasome activation induces increased intestinal permeability and that this is abrogated by LRRK2-kinase inhibition of NLRC4 inflammasome production of IL-1β but not IL-18 production. These studies thus highlight a mechanism by which LRRK2 inhibitors ameliorate gut inflammation.

## Results

### LRRK2 kinase inhibitors block LRRK2/NLRC4 interaction and NLRC4 phosphorylation at Ser533

In previous studies by Liu et al. (9) it was reported that in murine cells LRRK2 and NLRC4 interact with one another via their WD40/kinase domains and NBD/LRR domains, respectively; moreover, such interaction was partially inhibited by the LRRK2-kinase inhibitor GSK2578215A. In our initial studies, we re-examined such LRRK2/NLRC4 interaction and inhibition in human cells and found that in the latter cells, such interaction does indeed occur, but that in this case, inhibition of interaction was complete rather than partial. Thus, in studies of inhibition of interaction by two other LRRK2-kinase inhibitors, LRRK2-IN-1 and CZC54252.HCl, determined in both an HEK293t cell-LRRK2/NLRC4 over-expression system and in human PBMC-derived DCs stimulated with LPS and Needle-Tox (**Supplementary Figures 1A, B**), no interaction between LRRK2 and NLRC4 could be detected in the presence of inhibitors.

Liu et al. also provided evidence that in murine cells, phosphorylation of NLRC4 at Ser533 is marginally (but not significantly) increased above the baseline in *S. typhimurium*-stimulated murine LRRK2^-/-^ cells and not at all increased above baseline in LPS+Flagellin or LPS+PrgJ-stimulated cells. In re-examination of these findings in human cells, we found that the two inhibitors mentioned above efficiently inhibited NLRC4 phosphorylation at Ser533 in PBMC-derived DCs stimulated with LPS and Needle-Tox; in addition, these kinase inhibitors completely inhibited such phosphorylation in these cells stimulated with Needle-Tox alone or *S. typhimurium*, provided that they also completely inhibited LRRK2 phosphorylation, a measure of LRRK2 activation (**Supplementary Figures 1C-E**). Finally, DCs subjected to knock-out of LRRK2 mRNA by CRISPR-Cas9 editing (facilitated by lentivirus transduction) and then stimulated with *S. typhimurium* exhibited undetectable NLRC4 phosphorylation, again indicating the dependency of phosphorylation on LRRK2 and showing that inhibition of phosphorylation by LRRK2-kinase inhibitors was not due to off-target inhibition of other kinases (**Supplementary Figure 1F**).

Overall, the above data support the view that in human cells, LRRK2 must retain its kinase function to interact with NLRC4 and that, in these cells, LRRK2 is both necessary and sufficient for optimal NLRC4 phosphorylation.

### Inhibition of NLRC4 phosphorylation by LRRK2-kinase inhibitors reduces NLRC4 inflammasome IL-1β but not IL-18 generation in human PBDCs

**Figure 1:**
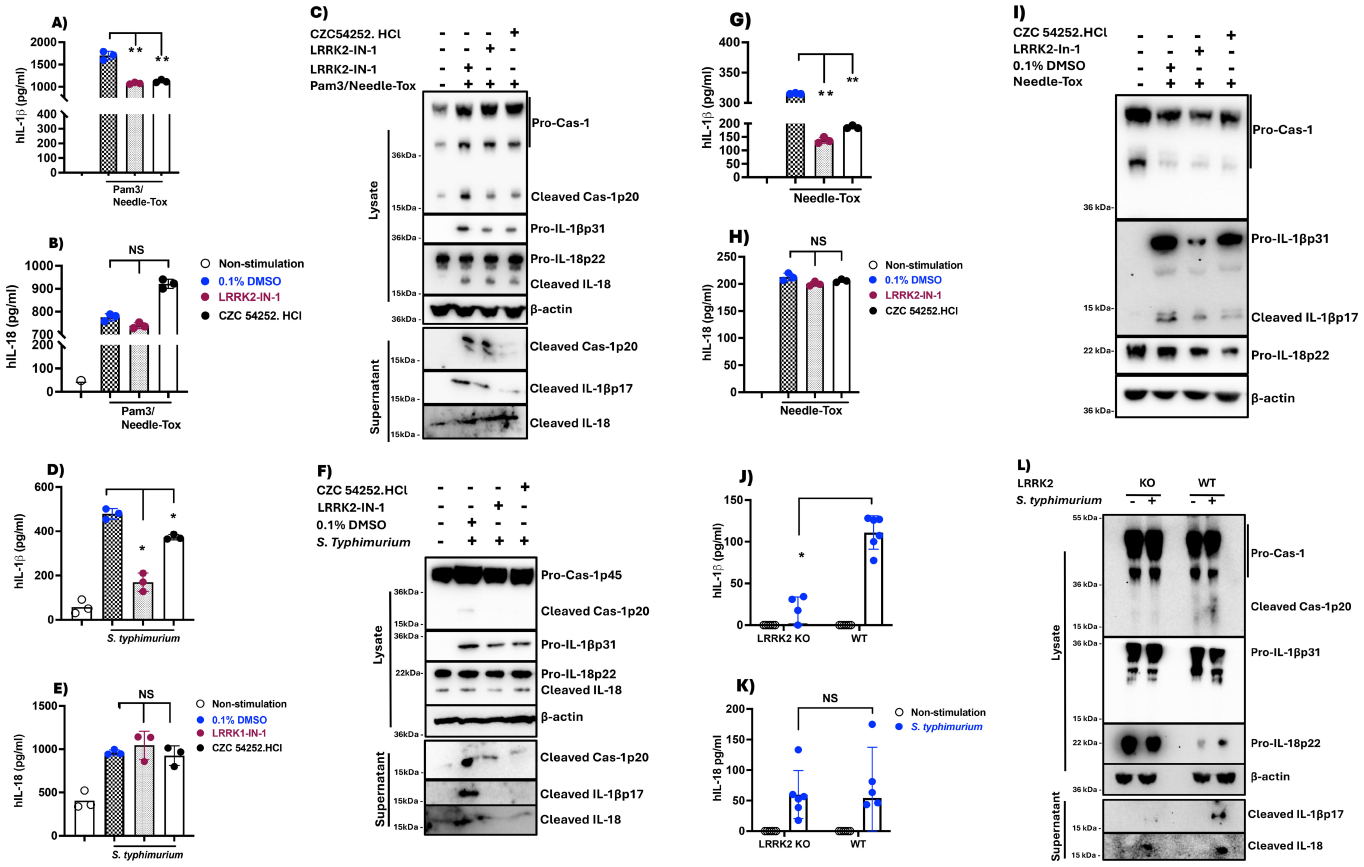
LRRK2-kinase inhibitors suppress IL-1β but not IL-18 generation in human PB-DCs. PBMC-derived dendritic cells, generated over 6 days as described in Methods, were harvested on day 7 and adjusted to a concentration of 1x10^6^ cells/ml. Cells were placed in 12-well culture plates at 1ml/well. The next morning, the media was replaced with fresh, complete RPMI media containing 0.1% DMSO alone or one of two LRRK2 inhibitors, LRRK2-IN-1 and CZC 54252.HCl at a concentration of 5 µM and treated for 60 minutes. Subsequently, cells were stimulated with TLR ligands and/or various NLRC4 ligands (or *S. typhimurium*) were added to the culture sequentially to obtain the indicated concentration for various lengths of time, after which culture supernatants were harvested and assayed for IL-1β and IL-18 levels by ELISA. Cell lysates derived from cells at the termination of treatment were subjected to immunoblot analysis using the indicated antibodies. **(A-C)** Pam3CSK4 (100 ng/ml, 3h), followed by Needle-tox (1 µg/ml, 30 minutes) was added to the culture; **(D-F)** *Salmonella typhimurium* (MOI-1, 4 h); **(G-I)** Needle-tox (1 µg/ml, 4h). **(J-L)** Human PBMC-derived dendritic cells were subjected to LRRK2 KO via CRISPR/Cas9 editing facilitated by lentivirus transduction as described in Methods and were cultured as indicated above with *Salmonella typhimurium* (MOI-1, 4 h). Blue dots indicate 0.1% DMSO + stimulant, Dark brown indicates LRRK2-IN-1+stimulant, and Black indicates CZC 54252.HCl. + stimulant. Data are shown as means ± SEM; *,P< 0.05; **,P< 0.01; ***,P< 0.0001; ****, P<0.00001; as determined by Student’s t-test. All conditions were tested in triplicate; in each panel, the data shown represent at least three (3) independent experiments.

The fact that LRRK2-kinase inhibitors cause complete rather than partial loss of NLRC4 phosphorylation at Ser533 in human cells allows one to utilize such inhibitors to more definitively explore the role of phosphorylation in NLRC4 inflammasome function, inasmuch as any lack of effect caused by LRRK2 inhibition cannot now be attributed to partial inhibition of NLRC4 phosphorylation. Accordingly, we cultured human PBMC-derived DCs (PBDCs) (as indicated in the Methods) with Pam3CSK4 (a TLR2 ligand) and Needle-Tox in the presence and absence of two LRRK2 inhibitors (LRRK2-in-1 and CZC54252.HCl), and then evaluated both IL-1β and IL-18 responses by ELISA and immunoblotting. We found that inhibition of LRRK2-kinase function by both inhibitors partially (but substantially) inhibited NLRC4 inflammasome-dependent IL-1β generation, but surprisingly, had no effect on IL-18 generation as evaluated by both ELISA assay of cytokine secretion and immunoblotting of cell lysates (**Figures 1A–C**). This mixed response was accompanied by a complete loss of LRRK2 phosphorylation and a complete loss of NRLC4 phosphorylation. Similar results were obtained in studies wherein PBDCs were stimulated by *S. typhimurium* (**Figures 1D–F**). Finally, we stimulated PBDCs with Needle-Tox in the absence of TLR-ligand co-stimulation to eliminate the possibility of confounding stimulatory effects unrelated to NLRC4 inflammasome activation and again found that the presence of LRRK2-kinase inhibitors led to complete inhibition of NLRC4 phosphorylation, accompanied by substantial and significant reduction of IL-1β production but little or no reduction of IL-18 production (**Figures 1G–I**).

In studies complementing the above LRRK2 inhibitor studies, we investigated NLRC4 inflammasome function in the absence of LRRK2 or in the presence of LRRK2 without kinase function. In the former case, we stimulated PBDCs in which LRRK2 expression had been substantially deleted by CRISPR-Cas9 editing (facilitated by lentivirus transduction) with S. typhimurium (MOI of 1.0) and found that cells lacking LRRK2 exhibited reduced IL-1β production but little or no reduction in IL-18 production upon NLRC4 inflammasome activation(**Figures 1J–L**). In the latter case, we transfected HEK293t cells with plasmids expressing NLRC4 inflammasome components as well as wild-type LRRK2 and LRRK2 with mutations known to cause enhanced kinase function (G2019S) or loss of kinase function (D2017A); we then cultured the transfected cells with or without one of two LRRK2 kinase inhibitors and determined their effect on the spontaneous NLRC4 inflammasome activation occurring in the transfected cells. We found that whereas the LRRK2-kinase inhibitors decreased NLRC4 inflammasome production of IL-1β, it had no effect on production of IL-18 in cultures containing wild-type or enhanced kinase function LRRK2; in addition, it had no effect on either IL-1β or IL-18 production in cultures containing kinase-dead LRRK2 (**Supplementary Figure 2**). Together, these studies support the above LRRK2 inhibitor studies in showing that the differential effect of inhibitors on IL-1β and IL-18 is, in fact, dependent on LRRK2 function and is not an inhibitor off-target effect.

Yet another explanation of the observed inhibitory LRRK2 effects on the NLRC4 inflammasome activation is that, at least with respect to IL-1β production, such effects are artifactually due to concomitant stimulation of NLRP3 inflammasome activation. To address this possibility, we cultured cells with/without MCC950 (3 µM, 30min) prior to stimulation with NLRC4 inflammasome ligands (Pam3CSK4 + Needle-Tox or Needle-Tox alone) or an NLRP3 inflammasome ligand (Pam3CSK4 + ATP) in the presence or absence of LRRK2 kinase inhibitors. The data obtained showed that MCC950 suppressed NLRP3 inflammasome activation but did not suppress NLRC4 inflammasome activation. In addition, MCC950 suppressed NLRP3 inflammasome-mediated IL-18 production but not NLRC4 inflammasome-mediated IL-18 production (**Supplementary Figure 3**). This result indicates that LRRK2-mediated effects on NLRC4 inflammasome activation are not due to effects on NLRP3 inflammasome activation.

Finally, it should be noted that, at least under TLR ligand-free conditions, the lack of effect of LRRK2-kinase inhibition on IL-18 production was not due to differential rates of IL-1β and IL-18 production or release since the time of culture supernatant harvest (at 4hr) was determined to be optimal for both cytokines in a time-course study (data not shown).

### NLRC4 phosphorylation in murine BMDCs is dependent on LRRK2-kinase activity and affects NLRC4 inflammasome IL-1β production but has little or no effect on its IL-18 production

**Figure 2:**
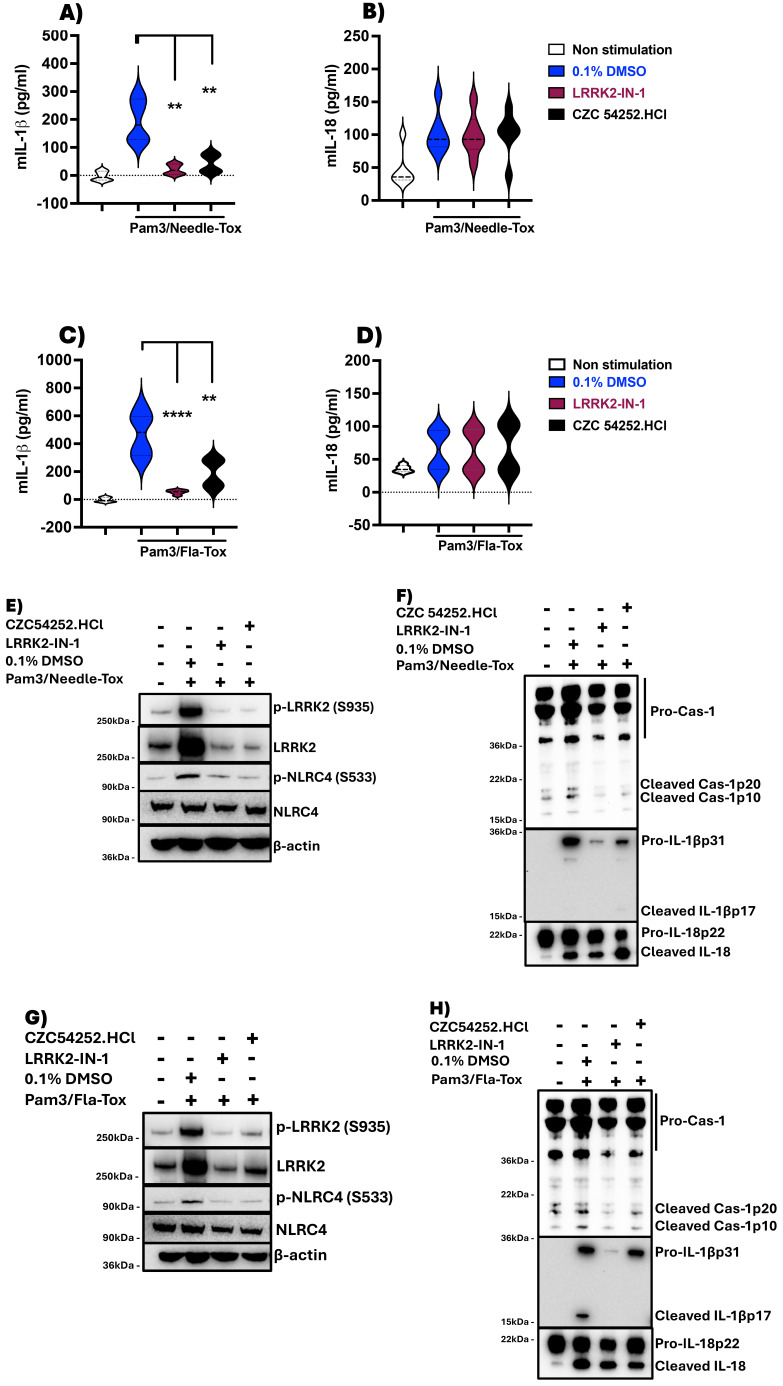
LRRK2-kinase inhibitors suppress IL-1β but not IL-18 production in murine BMDCs. Mouse BMDCs were generated over 6 days as described in Methods, harvested on day 7, and adjusted to a concentration of 1x10^6^ cells/ml. Cells were placed in a 12-well culture plate at 1ml/well. The next morning, the media was replaced with fresh, complete RPMI media containing 0.1% DMSO alone or one of two LRRK2 inhibitors, LRRK2-IN-1 and CZC 54252.HCl at a concentration of 5 µM and treated for 60 minutes; subsequently, various TLR ligands and/or various NLRC4 ligands were added to the culture sequentially to obtain the indicated concentration for various lengths of time, after which culture supernatants were harvested and assayed for IL-1β and IL-18 levels by ELISA. **(A, B)** Pam3CSK4 (100 ng/ml, 3h) followed by Needle-Tox (1 µg/ml, 30 minutes); **(C, D)** Pam3CSK4 followed by Fla-Tox (1 µg/ml for 60 minutes). **(E-H)** Cell lysates derived from cells at the termination of culture, which had been treated as described in **(A-D)**, were subjected to immunoblotting with the indicated antibodies. Blue violins indicate 0.1% DMSO + stimulant, Dark brown violins indicate LRRK2-IN-1+ stimulant, and Black violins indicate CZC 54252.HCl.+ stimulant. Data in **(A-D)** are shown as means ± SEM; *,P< 0.05; **,P< 0.01; ***,P< 0.0001; ****, P<0.00001; as determined by Student’s t-test. All conditions were tested in triplicate; in each panel, the data shown represent at least two independent experiments.

To determine if LRRK2 kinase affects NLRC4 phosphorylation and inflammasome function vis-à-vis IL-1β and IL-18 in the same manner in murine DCs as it does in human DCs, we evaluated murine bone marrow-derived DCs (BMDCs) responses in cultures containing either Pam3CSK4 plus Needle-Tox or Fla-Tox with or without the LRRK2-kinase inhibitors that prevented LRRK2-kinase activation (phosphorylation) in human DCs (**Supplementary Figures 1C-E**). We found that the LRRK2-kinase inhibitors did indeed inhibit NLRC4 phosphorylation in mouse BMDCs as described previously by Liu et al. (9) (**Figures 2E, G**). In addition, these findings were supported by studies of DCs derived from LRRK2^-/-^ mice, which showed that NLRC4 inflammasome activation was usually (but not always) accompanied by lack of NLRC4 phosphorylation (data not shown). This inconsistency was in line with the fact that long-exposure immunoblot analysis of DCs from LRRK2^-/-^ mice employing LRRK2 antibody with proven specificity revealed that these cells express low levels of LRRK2.

In related studies to determine the effect of absent or deficient NLRC4 phosphorylation caused by deficient LRRK2-kinase activity on NLRC4 inflammasome function we found that in the presence of LRRK2-kinase inhibitors, murine BMDC simulated by Pam3CSK4 and Needle-Tox or Pam3CSK4 and Fla-Tox displayed substantially and significantly reduced NLRC4 inflammasome generation of IL-1β but had little or no effect on generation of IL-18 (**Figures 2A–D, F, H**). Similarly, BMDCs from WT and LRRK2^-/-^ mice cultured with LPS plus Fla-Tox or Needle-Tox exhibited greatly and significantly decreased IL-1β production, whereas they exhibited, if anything, increased IL-18 production as compared to that in WT cells (**Supplementary Figures 4A, B**). These results support the conclusion that the LRRK2-kinase effect on NLRC4 phosphorylation is essential to the latter’s optimizing effect on IL-1β production but not on IL-18 production during NLRC4 inflammasome activation in murine cells, as well as in human cells.

### NLRC4 phosphorylation requires ASC incorporation into the assembling NLRC4 inflammasome

**Figure 3:**
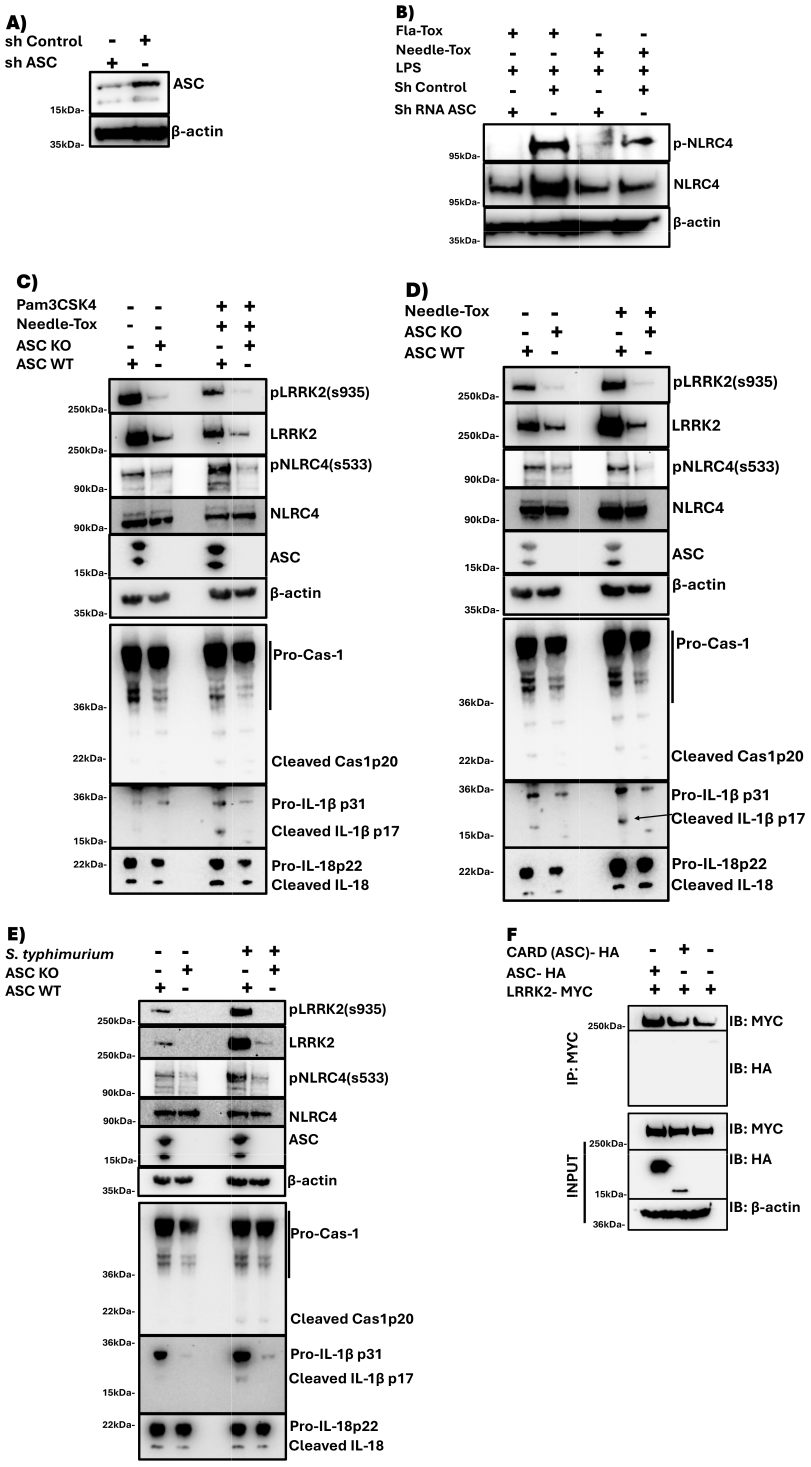
NLRC4 phosphorylation requires ASC incorporation into the NLRC4 inflammasome. **(A, B)** PBMC-derived DCs were subjected to ASC KD or sham KD by transfection with shRNA targeting ASC or scrambled shRNA, and cultured at 1×10^^6^/ml with LPS (200 ng/ml, 3 h) and Needle-Tox (1 μg/ml, 30 min). Afterward, cell lysates were obtained and subjected to immunoblotting as indicated. **(C-E)** ASC KO and WT THP-1 cells (1×10^^6^/ml) were separately cultured alone or with Pam3CSK4 (100 ng/ml, 3 h), followed by Needle-Tox (1 μg/ml, 30 min) **(C)**, with Needle-Tox (1 μg/ml, 3 h) alone **(D)**, or with *S. typhimurium* (MOI 1, 4 h) **(E)**. Afterward, cell lysates were collected and subjected to immunoblotting as indicated. **(F)** HEK293t cells were transfected with plasmids expressing MYC-tagged LRRK2, with or without co-transfection of plasmids expressing HA-tagged WT full-length ASC or ASC^CARD^ domain. After 24 hours, cell lysates were obtained and subjected to immunoprecipitation with anti-MYC antibody, followed by immunoblotting with the indicated antibodies. All the above data are representative of at least two independent experiments.

To further examine the role of LRRK2-mediated phosphorylation in NLRC4 inflammasome function, we next determined the relation of NLRC4 phosphorylation to ASC expression and function. Addressing the relation of phosphorylation to ASC expression first we explored the effect of ASC deletion on NLRC4 phosphorylation during NLRC4 inflammasome activation. Here we found that human PBDCs subjected to ASC knock-down with shRNA and then cultured with LPS plus Needle-Tox to induce NLRC4 inflammasome activation exhibited greatly diminished or absent NLRC4 phosphorylation compared to control cells without knock-down (**Figures 3A, B**). Similarly, we found that PBDCs subjected to ASC partial knock-out (KO) by CRISPR-Cas9 editing (facilitated by lentivirus transduction) and then cultured with Pam3CSK4 plus Needle-Tox or Needle-Tox alone exhibited greatly diminished NLRC4 phosphorylation (**Supplementary Figures 5A** and **5B**) compared to control cells. Finally, we found that THP1 ASC KO macrophages, i.e., cells with complete ASC deletion, when cultured with Pam3CSK4 plus Needle-Tox to induce NLRC4 inflammasome activation, expressed little or no NLRC4 phosphorylation whereas WT THP1 cells did express such phosphorylation (**Figure 3C**). Together, these data indicate that despite the fact that LRRK2 binds to NLRC4 in the absence of ASC (data not shown), incorporation of ASC into the assembling NLRC4 inflammasome is a necessary precondition for NLRC4 phosphorylation.

A possible explanation for the latter precondition came from studies showing that during activation of the NLRC4 inflammasome in ASC KO cells, LRRK2 remains in a non-phosphorylated and hence inactivated state that likely renders it unable to phosphorylate NLRC4 (**Figures 3D, E**). This finding implies that LRRK2 is itself activated during NLRC4 inflammasome activation and is only then capable of NLRC4 inflammasome activation. If this is indeed the case, however, it is not likely a result of direct ASC/LRRK2 interaction since we found no evidence that the latter occurs by immunoprecipitation/immunoblot study (**Figure 3F**).

**Figure 4:**
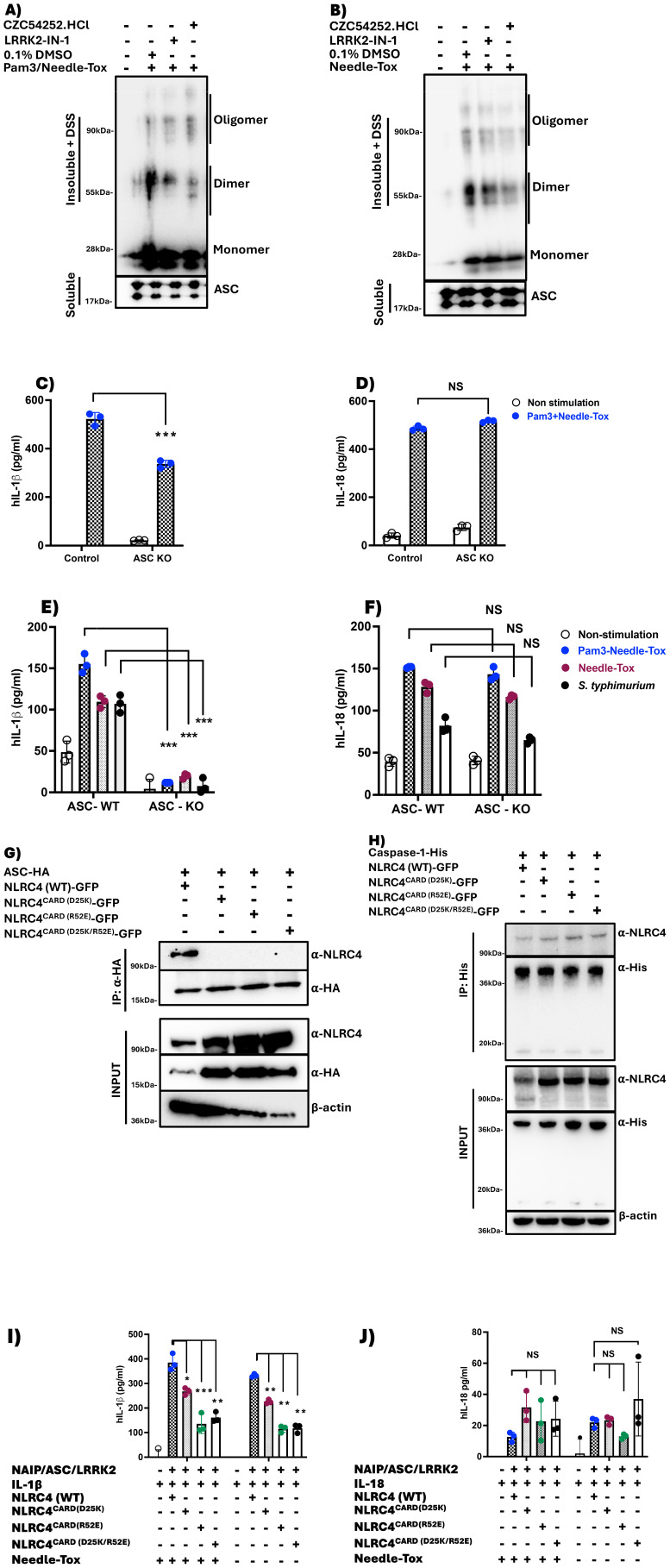
NLRC4 processing of pro-IL-1β and pro-IL-18 is differentially affected by ASC. **(A, B)** PBMC-derived DCs, generated as described in **Figure 1**, were cultured in media containing 0.1% DMSO alone or LRRK2 inhibitors (5 µM) for 60 min. Subsequently, cells were treated with Pam3CSK4 (100 ng/ml for 3 h) plus Needle-Tox (1 µg/ml for 30 min) or Needle-Tox alone (3h). At the end of culture, the cells were lysed using a lysis buffer, and the insoluble fraction obtained was cross-linked with DSS to capture ASC oligomers. ASC insoluble fractions (insoluble + DSS) and soluble fractions were subjected to SDS PAGE and detected with an antibody against ASC. **(C-D)**, Partial ASC KO in human PBDCs was performed by CRISPR-Cas9 editing facilitated by lentiviral transduction, as described in Methods, and stimulated as described in condition (**A**, without inhibitors). **(E-F)**, ASC KO THP- 1 cells were cultured under conditions (**A-B**, without inhibitors), in addition to *S. typhimurium* (MOI 1 for 4h). Cell culture supernatants collected at the end of culture were assayed for IL-1β and IL-18 levels by ELISA. **(G-H)**, HEK 293t cells were transfected with plasmids expressing HA-tagged ASC or His- tagged Caspase 1, co- transfected with or without GFP- tagged WT full- length NLRC 4 or NLRC 4 with one of three CARD domain mutations: NLRC 4 ^CARD (D 25 K)^, NLRC 4 ^CARD(R 52 E)^, and NLRC 4 ^CARD(D 25 K/R 52 E)^. After 24 hours, the cells were harvested and lysed, then subjected to immunoprecipitation with anti-HA **(G)**, or anti-His antibodies **(H)**, and immunoblotted with the indicated antibodies. **(I-J)**, HEK 293t cells were transfected with plasmids expressing NAIP, ASC, and LRRK 2, with or without GFP- tagged WT full- length NLRC4, or NLRC4 with one of three CARD domain mutations (NLRC 4 ^CARD (D 25 K)^, NLRC 4 ^CARD(R 52 E)^, and NLRC 4 ^CARD(D 25 K/R 52 E)^) as well as with plasmids expressing IL- 1 β **(I)**, or IL- 18 **(J)**, after 24 hours, they were stimulated with or without Needle-Tox (1 µg/ml) for an additional 24 hours, after which supernatants were collected and assayed for IL- 1 β and IL- 18 by ELISA. All results are representative of at least two independent experiments. Data are shown as means ± SEM; *,P< 0. 05; **,P< 0. 01; ***,P< 0. 0001. Statistical significance was determined using Student’ s t- test. All conditions were tested in triplicate.

We next addressed the relation of NLRC4 phosphorylation to ASC function. In previous studies, it was shown that ASC oligomerization during *S. typhimurium*-induced NLRC4 inflammasome assembly is significantly impaired in LRRK2^-/-^ cells, suggesting that ASC oligomerization depends on NLRC4 phosphorylation (9). In the present study, we showed the latter is in fact the case by demonstrating that ASC oligomerization during Pam3CSK4 plus Needle-Tox or Needle-Tox alone induction of NLRC4 inflammasome activation is significantly inhibited by various LRRK2-kinase inhibitors (**Figures 4A, B**). In addition, with studies of ASC speck formation during NLRC4 inflammasome activation by Needle-Tox alone, we showed that LRRK2-kinase inhibition impaired ASC speck formation (**Supplementary Figure 6**). These findings indicate that whereas ASC is necessary for NLRC4 phosphorylation, as shown above, such phosphorylation is, in turn, necessary for the ASC oligomerization and speck formation mediating its caspase cleavage function.

### NLRC4 processing of pro-IL-1β and pro-IL-18 is differentially affected by ASC

It is known that during NLRC4 inflammasome activation caspase can be activated and cleaved via a CARD/CARD interaction with dimeric (or oligomeric) ASC that is bound to NLRC4 (also via a CARD/CARD interaction); alternatively, caspase can be activated more directly via a direct CARD/CARD interaction with NLRC4 which is ASC independent (20, 21). With these mechanisms of NLRC4 inflammasome caspase activation in mind, we next determined how LRRK2-kinase inhibition and its negative effect on NLRC4 phosphorylation and ASC dimerization influence NLRC4 production of IL-1β and IL-18. To this end, we assessed IL-1β and IL-18 production in human PBDCs subjected to KO of ASC using CRISPR-Cas9 editing (facilitated by Lenti virus transduction) and then cultured these cells in the presence of Pam3CK4 plus Needle-Tox. We found that PBDCs subjected to ASC KO consistently exhibited reduced IL-1β production compared to PBDCs without ASC KO, whereas IL-18 production was not significantly different under these two conditions (**Figures 4C, D**). Similar results were observed in the ASC KO THP-1 cell line treated with Pam3CSK4 with Needle-Tox or Needle-Tox alone or *S. typhimurium* (**Figures 4E, F**). These data therefore provided functional evidence that IL-1β cleavage and production depend partly on caspase activation by ASC and partly on caspase activation by NLRC4 alone, whereas IL-18 cleavage and production depend only on the latter.

The above conclusion, holding that pro-IL-18 cleavage by the NLRC4 inflammasome is independent of ASC function, is supported by some but not all prior studies (see Discussion). We therefore sought additional, preferably molecular, evidence in favor of its validity. In pursuit of such evidence, we noted that whereas the CARD domain of NLRC4 serves as a platform common to both pathways of NLRC4 inflammasome-mediated pro-IL-1β/IL-18 cleavage, the site of interaction between ASC^CARD^ and NLRC4^CARD^ on the one hand and caspase^CARD^ and NLRC4^CARD^ on the other may be different. Furthermore, we noted that since this possible difference could reflect differential usage of the ASC^CARD^ and the NLRC4^CARD^ in the activation of caspase for IL-1β and IL-18 cleavage, it could provide a molecular mechanism that explains the differing effect of NLRC4 phosphorylation on IL-1β and IL-18 generation.

Acting on this possibility we created plasmids expressing NLRC4 with CARD domain mutations that, based on prior cryo-EM studies by Li et al., defining NLRC4 CARD domain interactions with the caspase CARD domain and the ASC CARD domain at a molecular level (20), could be used to identify if IL-1β and IL-18 utilize different caspase cleavage pathways to achieve the mature form. Accordingly, we evaluated NLRC4 inflammasome function in HEK293t cells mediated by transfected plasmids expressing ASC and plasmids expressing WT NLRC4 or NLRC4 bearing mutations (D25K) and (R52E) or a double mutation (D25K/R52E) in the NLRC4^CARD^ domain that would likely disrupt the ASC^CARD^-NLRC4^CARD^ interaction predicted to exist by Li et al. (20),. In initial co-immunoprecipitation studies, we showed that in lysates of HEK293t cells transfected with ASC and WT or mutated NLRC4, ASC binds to WT NLRC4 but not to mutated NLRC4; in contrast, caspase 1 binds to both WT and mutated NLRC4 (**Figures 4G, H**). With this information in hand, we next assessed IL-1β and IL-18 outputs by HEK293t cells transfected with NAIP-, ASC-, and LRRK2-expressing plasmids as well as an NLRC4-expressing plasmid bearing WT CARD domains or CARD domains with mutations specifically affecting ASC/NLRC4 CARD/CARD interactions, and then cultured the cells thus transfected with Pam3CK4 plus Needle-Tox. We found that production of IL-1β was significantly reduced whereas production of IL-18 was equal in cells transfected with mutated NLRC4-expressing plasmids as compared to cells transfected with WT NLRC4-expressing plasmids (**Figures 4I, J**). This result supports the view that IL-1β cleavage and secretion proceeds via both an indirect ASC^CARD^ domain/NLRC4^CARD^ domain interaction (that is disrupted by the mutation) and a direct caspase^CARD^/NLRC4^CARD^ interaction (that is not disrupted by the mutation); in contrast, IL-18 cleavage and secretion proceed solely via a direct caspase^CARD^/NLRC4^CARD^ interaction (that is not disrupted by the mutation).

### NLRC4 inflammasome activation and its inhibition by LRRK2 inhibitor in patients with Crohn’s disease

Patients with CD have two potential reasons to have increased NLRC4 inflammasome activity at sites of mucosal inflammation. The first is that these sites may contain increased concentrations of NLRC4 ligands arising from the appearance of gut bacteria that can express such ligands and inject them into hematopoietic cells or, alternatively, these sites may contain dying cells that release DAMPS that have been shown to have NLRC4 ligand properties such as lysophosphatidylcholine (LPC) and short interspersed nuclear elements (SINE) (16, 22). The second is that CD patients with intestinal inflammation may bear LRRK2 risk polymorphisms conferring increased LRRK2 expression and kinase activity or may simply express increased LRRK2 levels due to their exposure to pro-inflammatory cytokines (1) and, as shown by Liu et al., such increased expression/activity is associated with increased NLRC4 inflammasome activation in a re-constituted HEK293t cell system (9).

**Figure 5:**
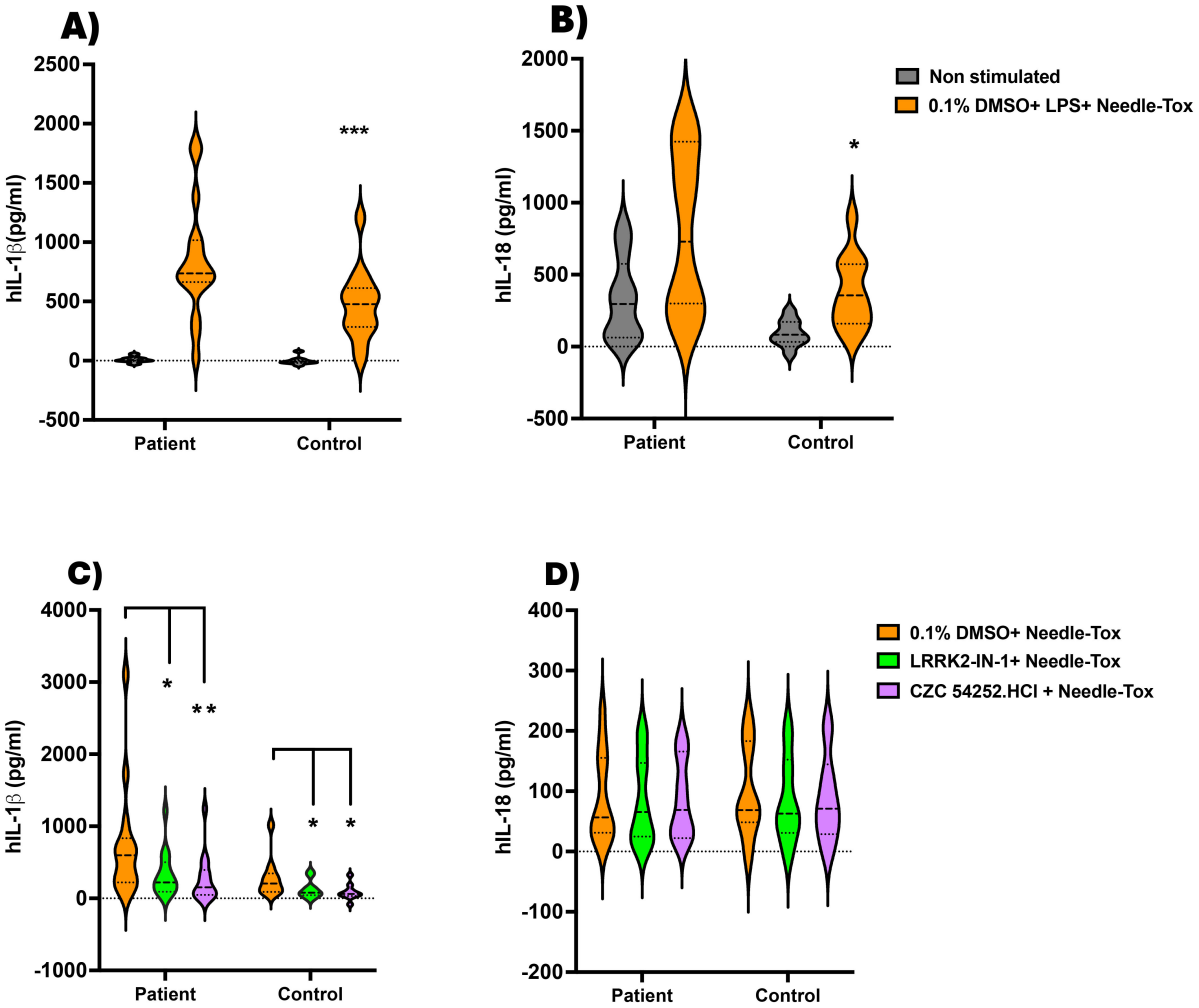
Inhibition of NLRC4 inflammasome activation by LRRK2 inhibitors in patients with Crohn’s disease. On the day before the experiment, PBMC-derived DCs (PBDCs) generated (as described in Methods) from PBMCs of patients with Crohn’s Disease (CD) (n=15) and healthy controls (n=13) were cultured at 1x10^^6^ cells/ml in 12-well plates containing 1 ml per well. **(A, B)** On the next morning, the culture medium was replaced with fresh complete RPMI medium, and the cells were stimulated with LPS (200 ng/ml, 3 h) and Needle-Tox (1 µg/ml, 30 min). ELISA analyzed cell culture supernatants at the end of the culture period to assess IL-1β and IL-18 levels. **(C, D)** 1x10^6^ PBMC/ml from CD patients (n=14) and healthy controls (n=10) were cultured as in **(A, B)**, with or without LRRK2 inhibitors LRRK2-IN-1 and CZC 54252.HCl (5 µM, 60 min), followed by Needle-Tox alone (1 µg/ml, 4 h). At the end of the culture period, cell culture supernatants were collected and analyzed by ELISA to assess IL-1β and IL-18 levels. Data are shown as means ± SEM; *,P< 0.05; **,P< 0.01; ***,P< 0.0001; as determined by Student’s t-test; all conditions were tested in triplicate.

In an initial study to examine NLRC4 inflammasome activation in CD, we cultured PBDCs from patients and controls with LPS plus Needle-Tox as indicated in the Methods. We found that IL-1β production in this group varied considerably, possibly because the patients studied consisted of individuals on therapy with varying levels of disease activity (**Supplementary Table 1**). Despite this variation, however, the patient mean NLRC4 inflammasome-mediated IL-1b and IL-18 responses exhibited by patients were significantly higher than those of the control responses (**Figures 5A, B**). These data thus indicate that Crohn’s inflammation is accompanied by the generation (and release into the circulation) of cells that are primed for NLRC4 inflammasome responses, presumably by exposure to NLRC4 ligand and/or the presence of increased LRRK2 kinase activity.

In a second and independent study, we cultured PBMC from patients with Crohn’s disease with Needle-Tox alone in the absence and presence of the LRRK2-kinase inhibitors, LRRK2-IN-1 and CZC54252.HCl, as indicated in Methods. This method of NLRC4 inflammasome stimulation minimizes confounding effects of concomitant TLR stimulation, albeit at the expense of response magnitude. The patient group studied again consisted of individuals on therapy with varying levels of disease activity and again yielded highly variable responses (**Supplementary Table 2**). In this case, we found that the mean level of IL-1β and IL-18 production by CD patient cells was not significantly greater than that of cells from control individuals, although the high producers were clearly more common in the patient group than in the control group. In addition, we found that IL-1β production by CD patient cells was significantly inhibited by each of the two LRRK2-kinase inhibitors, LRRK2-IN-1 and CZC54252.HCl, (**Figures 5C, D**); in contrast, mean IL-18 production by CD patient cells was not lowered by the presence of LRRK2-kinase inhibitor. Thus, whereas in the absence of TLR stimulation, CD patient cells did not manifest increased mean NLRC4 inflammasome activity, these cells did exhibit the differential impact of inhibition of LRRK2 on IL-1β and IL-18 production shown above in studies of cells from control individuals.

### Increased intestinal permeability induced by the NLRC4 inflammasome is inhibited by LRRK2-kinase antagonist

**Figure 6:**
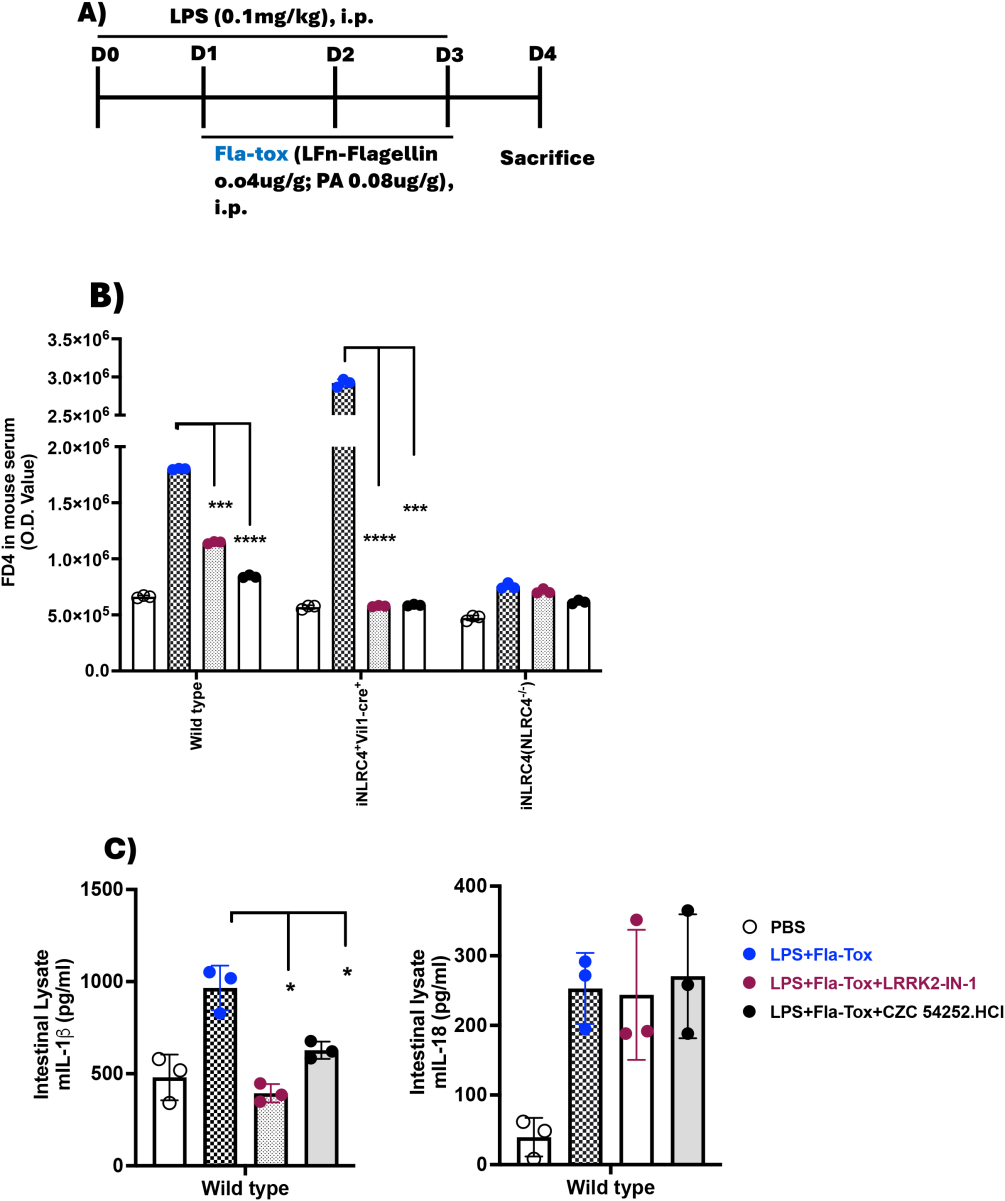
Increased Intestinal permeability induced by NLRC4 inflammasome activation is suppressed by LRRK2 inhibitors. **(A)** 8–10 week-old C57BL/6 WT, in-house iNLRC4^+^Vil1-cre^+^ (NLRC4 present only in epithelial cells), and NLRC4^-/-^ (iNLRC4) mice (n=3 per group) were administered PBS, LPS, Fla-Tox, and LRRK2 inhibitors CZC54252 and LRRK2-IN-1 at a dose of 5 mg/kg via intraperitoneal injection on days 0-3, following the protocol shown; **(B)** On day 4, mice were fasted for one hour, then given FD4 by gavage (10 mg/ml, 150 µl/mouse). After one hour, mice were euthanized to collect blood and intestinal tissue; the FD4 concentration in the blood serum was measured by fluorescence. Serum was pooled, and FD4 fluorescence was recorded. **(C)** Right Panel: IL-1β; Left Panel: IL-18. The entire intestine from mice treated according to the described protocol was homogenized, centrifuged, and the clear lysates were used to measure IL-1β and IL-18 levels by ELISA. Each dot represents one mouse. Blue dots indicate LPS+ Fla-Tox, Dark brown dots indicate LPS+ Fla-Tox + LRRK2-IN-1 and Black dots indicate LPS+ Fla-Tox +CZC 54252.HCl. Data are expressed as means ± SEM;*,P < 0.05; **,P < 0.01; ***, P< 0.0001; ****, P<0.00001; Both Student’s t-test or one-way ANOVA determined significance. All conditions were performed in triplicate. In each panel, data are from at least two independent experiments.

Inasmuch as the inhibition of the positive effect of LRRK2-kinase on NLRC4 phosphorylation by an LRRK2 inhibitor has only a partial effect on NLRC4 inflammasome IL-1β production and little or no effect on inflammasome IL-18 production, it was important to determine if this effect still has an impact on gut inflammation, such as that occurring in CD. We therefore determined the effect of LRRK2-kinase inhibition on epithelial barrier function, a function that might be particularly sensitive to NLRC4 inflammasome activity, given the fact that this inflammasome is highly active in epithelial cells in response to enteric pathogens. Accordingly, we administered LPS and Fla-Tox to mice by IP injection according to the protocol diagrammed in **Figure 6A** and measured intestinal permeability by FITC-dextran (FD4) serum level on day 4 of the regimen. We found that such administration significantly augmented intestinal permeability as measured by serum FD4 concentration in WT mice but not in either NLRC4^-/-^ or LRRK2^-/-^ mice, suggesting that LRRK2-kinase augmentation of NLRC4 inflammasome function is necessary for the induction of increased intestinal permeability by an NLRC4 ligand (**Supplementary Figures 7A–C**). Next, we determined the effect of LRRK2-kinase inhibitors on NLRC4 inflammasome-induced increased intestinal permeability using the same protocol, but in this case in WT, NLRC4 mice expressing NLRC4 only in epithelial cells (NLRC4^+VilCre^ mice), and in NLRC4^-/-^ mice. We found that whereas WT and NLRC4^+VilCre^ mice exhibited increased FD4 absorption, NLRC4^-/-^ did not, indicating that activation of the NLRC4 inflammasome was necessary for the observed increase in permeability, and moreover, such activation in epithelial cells was sufficient for the observed increase (**Figure 6B**). In addition, we found that LRRK2-kinase inhibitors significantly diminished the increased intestinal permeability (as measured by serum FD4 concentration) in WT and NLRC4^+VilCre^ mice and that in WT mice, this diminution was accompanied by decreased intestinal IL-1β production but not IL-18 secretion (**Figure 6C**). Thus, the partial inhibitory effect of LRRK2-kinase inhibition on NLRC4 inflammasome IL-1β production, accompanied by a lack of inhibition of IL-18 production, is sufficient to inhibit the potentially important pathologic effect of NLRC4 inflammasome activation, namely its effect on intestinal permeability.

## Discussion

In this study we define the role of NLRC4 phosphorylation in the regulation of NLRC4 inflammasome activation. First, we expand on prior studies by Liu et al. by showing that NLRC4 phosphorylation by activated LRRK2-kinase in human and very likely in murine mononuclear cells as well is both necessary and sufficient for optimal NLRC4 phosphorylation. Second, we demonstrate that the LRRK2-induced phosphorylation requires prior ASC association with the nascent NLRC4 inflammasome in human cells and indeed is necessary (as noted previously) for ASC polymerization, speck formation and caspase cleavage function. Finally, we show that LRRK2-kinase induced NLRC4 phosphorylation affects downstream IL-1β cleavage but has little or no effect on IL-18 cleavage, reflecting the fact that whereas IL-1β cleavage is partially dependent on ASC, IL-18 cleavage is largely independent of ASC. These findings explain the fact that, whereas NLRC4 inflammasome production of both IL-1β and IL-18 is increased in peripheral cells of patients with Crohn’s inflammation (who usually exhibit increased LRRK2 expression), IL-1β production is reduced in the presence of an LRRK2-kinase inhibitor, whereas there is little, if any, effect on IL-18 production.

ASC is essential to the activation of the NLRP3 inflammasome inasmuch as NLRP3 lacks a CARD domain and thus relies on its recruitment of the ASC pyrin domain to cleave caspases. Not so the NLRC4 inflammasome, which has its own CARD domain and can cleave caspases in the absence of ASC. The latter fact was verified here with studies in which we showed that activation of the NLRC4 inflammasome in PBDCs with ASC deletion still resulted in substantial IL-1β generation, albeit reduced as compared to that exhibited by cells without deletion. This bipartite capacity of the NLRC4 inflammasome to cleave caspase provides an explanation of why inhibition of NLRC4 phosphorylation has only a partial inhibitory effect on IL-1β generation because, as shown here [and in part by Liu et. al (9)], while NLRC4 phosphorylation is necessary for ASC oligomerization and its caspase cleavage function, the NLRC4 CARD domain is functionally capable of interacting with and cleaving caspase in the absence of phosphorylation and ASC caspase cleavage function. This bipartite capacity to cleave caspase also explains why IL-18 generation is unaffected by the absence of NLRC4 phosphorylation and ASC oligomerization, since one can now postulate that IL-18 cleavage is independent of ASC interaction with caspase and relies solely on the NLRC4/caspase interaction. To verify this possibility we took advantage of previous cryo-EM studies by Li et al. (20) in which NLRC4 CARD domain interactions with caspase CARD domain and ASC CARD domain were defined at a molecular level and which therefore pointed to the construction of NLRC4 deletion mutants that could identify the independence of ASC and NLRC4 cleavage of caspase with respect to IL-1β and IL-18. Acting on these prior cryo-EM studies, we performed transfection studies with NLRC4^CARD^ domain mutants (D25K, R52E, and double mutant, D25K/R52E) predicted to disrupt ASC^CARD^ NLRC4^CARD^ interaction and found that, indeed, these mutations rendered NLRC4 unable to interact with ASC but still able to interact with caspase 1. In addition, we found that the NLRC4 inflammasome with these mutations were still able to generate cleaved IL-1β (albeit at a reduced level compared to unmutated NLRC4) and also to generate cleaved IL-18 at the same level as unmutated NLRC4. These studies thus provide strong additional evidence that IL-18 is unaffected by lack of NLRC4 phosphorylation because its cleavage into a mature form is independent of ASC-mediated interaction with caspase.

The above conclusion is supported by several previous studies showing that IL-18 secretion resulting from NLRC4 inflammasome activation in hematopoietic cells from mice with ASC deletion is intact and equal to that of cells without deletion (23, 24). In addition, it is supported by the fact that regulation of IL-1β and IL-18 cleavage and secretion by the NLRC4 inflammasome appear to be separately regulated in that mutations in NLRC4 lead to differential effects on IL-1β and IL-18 secretion and isolated excess of IL-18 secretion (25, 26). On the other hand, there are also reports in which it was found that cells from mice with ASC deletion exhibit reductions in IL-18 secretion (27, 28). These contrary findings, however, are tempered by the fact that activation of the NLRC4 inflammasome has been shown to lead to interaction with and activation of the NLRP3 inflammasome (29); in addition, NLRC4 ligands are promiscuous in that they can directly co-activate the NLRP3 inflammasome (30). Thus, it is likely that studies purporting to show that ASC is essential to NLRC4 inflammasome-mediated IL-18 cleavage, particularly those that use whole organisms at high concentrations as activators, are actually demonstrating the role of ASC in NLRP3 inflammasome IL-18 cleavage, rather than its role in NLRC4 inflammasome IL-18 cleavage. The studies shown here, conducted with specific NLRC4 ligands or low doses of whole organisms, and reflecting the effects of inhibition that only apply to NLRC4 inflammasome activation, are less subject to this critique.

The regulation of NLRC4 inflammasome responses by LRRK2-mediated phosphorylation discussed above was explored in PBDCs of patients with CD, an intestinal inflammatory disease previously shown to be associated with elevated LRRK2 levels (31). Two separate studies were conducted, using two different conditions for NLRC4 inflammasome activation. In the first study, cells were stimulated with a TLR ligand, LPS, plus an NLRC4 ligand, Needle-Tox, to best assess *in vivo* conditions of NLRC4 inflammasome activation. In the second study, cells were stimulated with an NLRC4 ligand in the absence of TLR stimulation to minimize possible confounding effects of stimulation of inflammasomes other than the NLRC4 inflammasome. One limitation in the results obtained from both of these studies relates to the fact that the patient cohorts varied in severity of disease and treatment regimen; this probably accounts for the wide variability in the magnitude of the responses. Another limitation was that the studies were conducted with circulating cells that may not fully mimic the function of cells at sites of inflammation. Despite these limitations, the results of first study were clear in showing that circulating cells from patients with Crohn’s disease exhibited significantly higher mean NLRC4-induced IL-1β and IL-18 responses than cells from control individuals. This probably reflects the fact that the patient cells studied originate (at least in part) from inflammatory sites that are enriched in NLRC4 ligands and have therefore been “primed” at these sites to mount increased NLRC4 responses. In addition, the increased responses could be due to increased LRRK2 levels or state of activation at intestinal sites, particularly in those patients bearing LRRK2 risk polymorphisms that have been shown to be associated with increased LRRK2 expression. In the second study, culture of patient cells stimulated by NLRC4 ligand alone in the presence of LRRK2-kinase inhibitors exhibited significantly reduced NLRC4 inflammasome production of IL-1β but little or no reduction in production of IL-18. Of some interest, the inhibitory effects of the inhibitor on IL-1β production, while significant, were less apparent in control cells than in patient cells, possibly due to the fact that the lower level of NLRC4 inflammasome activation and cytokine production observed in the latter cells is less dependent on NLRC4 phosphorylation and ASC-mediated caspase cleavage. In any case, this study of patient cells provides ample verification of the relation of NLRC4 phosphorylation to NLRC4 inflammasome function that was previously observed in detailed studies of cells from control individuals or stable cell lines.

The higher levels of NLRC4 inflammasome activity in patients with Crohn’s disease noted in our studies prompts a consideration of the role of this inflammasome in Crohn’s disease pathogenesis. Here it should be mentioned first that whereas IL-1β levels are increased in Crohn’s disease and, as such, are part of the potent pro-inflammatory cytokine package driving this inflammation, it has not been established that this cytokine plays an indispensable pro-inflammatory role in most patients. This view derives from the fact that despite the existence of agents that efficiently block IL-1β activity there are no published studies showing that these agents have therapeutic value in the ordinary patient with Crohn’s disease (11). In addition, genetically-determined increases in NLRP3 inflammasome activity in humans or mice due to NLRP3 gain-of-function mutations are not usually associated with Crohn’s disease-like inflammation, possibly because in the presence of increased NLRP3 function, they are accompanied by activation of immune suppressor mechanisms (12). A contrasting view of IL-1β inflammatory activity in Crohn’s disease, however, emerges from the fact that several genetic abnormalities in humans causing increased IL-1β production have been described that do, in fact, lead to Crohn’s disease inflammation, presumably in spite of possible compensatory mechanisms (11). In addition, there is evidence that IL-1β secretion, possibly from atypical cellular sources such as fibroblasts, contribute to severe, treatment unresponsive inflammatory bowel disease not associated with a mutation (32). On this basis, it is possible or even likely that NLRC4 inflammasome activity, particularly when augmented by the presence of a particular genetic and/or environmental factor, can make an important contribution to CD-associated inflammation and its inhibition by LRRK2-kinase inhibition plays an important role in its overall ability to treat Crohn’s disease.

One reason to propose that the NLRC4 inflammasome may be functioning at an increased level in at least some patients with Crohn’s disease is that the latter are exposed to non-pathologic organisms that express proteins that are NLRC4 inflammasome ligands. Relevant here is that whereas gram-negative Proteobacteria organisms capable of expressing a major NLRC4 ligand, flagellin, are typically present in low numbers in healthy individuals (33–35), such bacteria (particularly the bacterial species in the E. coli genus) are increased in Crohn’s disease patients (36–38). In addition, AIEC (adherent/invasive E. coli) are frequently found in patients with Crohn’s disease and have been implicated in intestinal inflammation via their capacity to simulate the NLRC4 inflammasome (39). E. coli expressing an NLRC4 ligand (such as flagellin) may also possess the requisite mechanism of ligand insertion. This is suggested by a report that non-pathogenic E. coli strains can be found in the intestinal microflora of mice with DSS-colitis or humans with Crohn’s disease that induce the accumulation of inflammatory macrophages in intestinal tissues via NLRC4 activation (14). Finally, it has recently become apparent that the NLRC4 inflammasome is not actually dependent on the presence of intestinal organisms expressing and delivering NLRC4 ligands. This possibility arises from the observation that certain DAMPS, such as lysophosphatidylcholine and short interspersed nuclear element RNA, can also activate the NLRC4 inflammasome via atypical mechanisms (40). Thus, the inevitable cell death and release of potential NLRC4 ligands occurring during Crohn’s inflammation can also result in NLRC4 inflammasome activation even in the absence of stimulating commensal or pathogenic organisms.

While, as suggested above, the most immediate reason for the increased NLRC4 inflammasome activation during Crohn’s inflammation is increased exposure of cells to NLRC4 inflammasome ligands, a second reason is also likely to be at play. This second reason is that the increased LRRK2 levels present in inflamed CD tissue resulting from IFN-γ (and possibly TNF-α) induction (31) is also a possible stimulus for increased NLRC4 inflammasome activation. This possibility was first supported by studies showing that NLRC4 inflammasome activation is augmented in HEK293t cells expressing LRRK2 with a kinase gain-of-function mutation (G2019S) (9) and later by studies showing that mice bearing LRRK2 with the same mutation exhibit more severe DSS-colitis than WT mice that is sensitive to LRRK2 kinase inhibition (41). The DSS-colitis in the latter LRRK2 KI mice was accompanied by increased IL-1β and IL-18 levels and was ameliorated by gasdermin D inhibition, together indicating that the increased inflammation was due to augmented inflammasome activity. Interestingly, these KI mice also manifested increased GI tumor development in the DSS/azoxymethane tumor model suggesting that increased LRRK2 levels and kinase activity may unleash both increased gut inflammation and tumorigenesis.

One way that the NLRC4 inflammasome and its augmentation by LRRK2 could have a particular effect on Crohn’s inflammation is related to the fact that this inflammasome is present in gut epithelial cells and is known to play a key role in host defense against infection with Salmonella (and perhaps other pathogens) via induction of epithelial cell-related organism expulsion (42). In relation to this NLRC4 inflammasome function, we showed that parenteral activation of the NLRC4 inflammasome by parenteral administration of ligand causes increased intestinal permeability that is negated by co-administration of LRRK2 inhibitor. Moreover, this effect can be mediated by NLRC4 inflammasome activity in epithelial cells as well as cells in the lamina propria, since it is seen in mice that only express NLRC4 in epithelial cells and not in NLRC4 KO mice. These studies thus suggest that whereas the direct contribution of the NLRC4 inflammasome to Crohn’s inflammation is yet to be determined, its indirect effect on the latter, owing to its strategic location in epithelial cells, may endow this inflammasome with unexpected pro-inflammatory importance. In addition, this role of the NLRC4 inflammasome in gut inflammation bolsters the possible use of an LRRK2 inhibitor as an effective treatment for Crohn’s disease inasmuch as the inhibitor blocks the detrimental effect of IL-1β on epithelial permeability while it preserves the support of IL-18 in epithelial barrier function (43).

## Materials and methods

### Human subjects

Blood samples to be studied were collected from Crohn’s disease patients at Mount Sinai Hospital in New York following acquisition of informed consent and then shipped on ice overnight to the NIH Clinical Center. Patient disease activity, defined by their CRP and ESR values, treatment profile, and other clinical information, was determined at the time of blood collection (**Supplementary Tables 1**, **2**). Control blood samples to be studied were collected from healthy volunteers at Mount Sinai Hospital; these individuals had no history of IBD or infectious disease at the time of the blood collection. In addition, other cells were obtained from Lymphopac apheresis specimens prepared by the NIH Blood Bank under established/approved procedures. The Institutional Review Boards of each Institution approved study protocols covering blood collection.

### Mice

C57BL/6J (Jax 000664) and Lrrk2 KO (JAX012453) mice were purchased from The Jackson Laboratory. iNLRC4^+^Vil1-cre^+^ and iNLRC4^-/-^ mice were gifts from Dr. Isabella Rauch, Oregon Health and Science University (42). 7-8-week-old mice were used for all experiments. Upon completion of the study, mice were anesthetized using 100 μL isoflurane (McKesson, USA) and euthanized by cervical dislocation. At times, a blood sample was collected by cardiac puncture prior to such euthanization. The Animal studies were conducted under NIH Animal Care and Use Committee-approved Protocols and approved NIH animal care guidelines.

### Reagents and cell stimulation

Recombinant LFn-flagellin and LFn-needle proteins were obtained from LifeSct, LLC (USA). These proteins were produced using a mammalian expression/secretion system, in which HEK293t cells were transfected with expression plasmids (pcDNA3.1(+)) encoding the respective sequences of genes related to these proteins, as reported by Rauch et al. (33). The recombinant proteins were subsequently purified from transfected HEK293t cells. Anthrax protective antigen (PA 63) (cat# 174) was sourced from Listlabs, CA, USA; LPS and Pam3CSK4 were obtained from InvivoGen, USA; and FD4 (Fluorescein isothiocyanate-dextran) was obtained from Sigma-Aldrich, USA. Antibody and reagent lists are described in **Supplementary Tables 2** and **3**.

For LRRK2-kinase inhibition, 1X10^6^ cells in culture plate wells were treated with LRRK2-kinase inhibitors at a concentration of 5 µM for 60 minutes.

For inflammasome activation, 1X10^6^ cells in culture plate wells were primed with either LPS (200 ng/ml, 3 hours) or Pam3CSK4 (100 ng/ml, 3 hours), followed by stimulation with Needle-tox or Fla-tox; the latter consisted of stimulation with a combination of recombinant LFn-Needle (1 µg/ml) or LFn-Flagellin (1 µg/ml) antigens, each in combination with anthrax protective antigen (1 µg/ml). Alternatively, cells were stimulated with *Salmonella typhimurium* (ATCC, Manassas, VA) added at a multiplicity of infection (MOI) of 1 for 4 hours.

### Human PBMC isolation and generation of DCs

PBMC were isolated from Lymphopac apheresis specimens (NIH blood bank) or whole blood using density-gradient centrifugation with LSM Lymphocyte Separation Medium (MP Biomedicals) and were washed twice with PBS. The cells were resuspended in RPMI 1640 complete medium supplemented with 20% FBS and 1% penicillin/streptomycin. 3X10^7^ cells/ml were seeded in a 100 cm² tissue culture plate (Corning, USA) along with 20 ng/ml of GM-CSF and 20 ng/ml of IL-4 (PeproTech, USA). The following day, floating cells and media were replaced with fresh RPMI complete medium supplemented with GM-CSF and IL-4. On day 4, half of the medium was replaced. On day 7, the attached cells were harvested and washed with PBS. 1X10^6^ cells/ml were resuspended in RPMI complete medium, and 1 ml of culture was plated in a 12-well plate (Corning, USA). The next day (day 8), the medium was changed and used for the experiment.

### Bone marrow-derived dendritic cells culture and inflammasome activation

Mouse femurs and tibias were flushed with a 23G needle and PBS (Gibco). Bone marrows were pelleted by centrifuging at 1500 rpm for five (5) minutes at 4C. Pellets were resuspended with 1ml ACK lysis reagent (Gibco) by pipetting vigorously, and adding 9ml PBS, pelleted again. Resuspend this pellet with complete medium (RPMI 1640 medium with 10% FBS and penicillin/streptomycin (Gibco)) in the presence of 20ng/ml of GM-CSF (PeproTech) and 20ng/ml of IL-4 (PeproTech) and cultured in a 100 cm^2^ petri dish. Every day 3, half of the medium was changed. On day 7, cells were harvested and washed with PBS, resuspended in a complete medium with a cell density 1X10^6^/ml, and plated 1ml/well in a six-well plate. On the next day, replace the medium with fresh complete medium and incubate the cells with either LPS (200 ng/ml) or Pam3CSK4 (100 ng/ml) for three hours, along with Fla-tox or needle-tox for an additional 60 or 30 minutes, respectively.

### Maintenance of cell lines

The THP-1 cell line was obtained from the American Type Culture Collection (ATCC), and the ASC knock-out THP-1 cell line (#thp-koascz) was sourced from InvivoGen, USA. These cells were maintained in RPMI 1640 medium supplemented with 10% FBS and 1% penicillin/streptomycin (Gibco). An additional 100 µg/ml of Zeocin™ and 50 µg/ml of Normocin™ (InvivoGen, USA) were added to the medium to cultivate the ASC knock-out cells. Cells were passaged every third day, and cell passages did not exceed 10 times. HEK293t cells were grown in DMEM/Glutamax (Life Technologies) supplemented with 10%FCS and penicillin/streptomycin (Gibco).

### shRNA transduction

ASC silencing was achieved using a short-hairpin RNA (shRNA)-based transfection method. The ASC shRNA plasmid (sc-37281) for human cells and the control shRNA plasmid (sc-108080) from Santa Cruz Biotech, USA, were obtained. 3X10^7^ PBMC/ml were seeded in a 100 cm² tissue culture plate (Corning, USA) along with 20 ng/ml of GM-CSF (PeproTech) and 20 ng/ml of IL-4 (PeproTech). The following day, the cell suspension and media were replaced with fresh RPMI complete medium, along with GM-CSF and IL-4. On day 3, the attached cells were harvested and washed with PBS. 2X10^6^ cells/ml were resuspended in RPMI (without any supplement), and 0.5 ml of culture (1X10^6^ cells/well) was plated in 6-well plates and transfected with 0.5 µg of plasmid using Lipofectamine LTX (ThermoFisher Scientific) diluted with Opti-MEM (ThermoFisher Scientific). Six (6) hours later, 0.5 ml of RPMI complete medium supplemented with 20 ng/ml of GM-CSF and 20 ng/ml of IL-4 was added. Seventy-two (72) hours later, the cells were harvested, washed with PBS, and used for the experiment.

### Lentiviral transduction and CRISPR editing

gRNA for LRRK2 (5’-3’: AGCGTTGACGATAAGCATT) (Sigma Aldrich) and ASC (5’-3’: TTGGACCTCACCGACAAGC) (Sigma Aldrich) were inserted into lentiCRISPR v2 (addgene # 52961) following the online LentiCRISPRv2 and LentiGuide-Puro protocols. Cloned plasmids were amplified using an endotoxin-free midi-prep kit (Qiagen). To produce lentivirus, gRNA-cloned lentiCRISPR v2 plasmids were co-transfected with packaging plasmids gag-pol, rev-tat, and VSV-G (44) into HEK293t cells. For virus collection, cell culture supernatants were harvested daily for three days, filtered (0.22 µm, EMD Millipore), and concentrated using a 100 kDa MWCO centrifugal concentrator (Millipore Sigma). The supernatant was concentrated tenfold, reducing 60 ml of supernatant to 6 ml.

For viral transduction, 3X10^7^ PBMC/ml were seeded in a 100 cm² tissue culture plate (Corning, USA) containing 20 ng/ml of GM-CSF (PeproTech) and 20 ng/ml of IL-4 (PeproTech). The following day, the floating cells and media were replaced with fresh RPMI complete medium supplemented with GM-CSF and IL-4. On day 3, the attached monocytes were harvested, and a 2X10^6^/ml cell suspension was prepared using one parts RPMI medium and one parts lentivirus-containing supernatant along with polybrene (1 µg/mL, Millipore Sigma), 20 ng/ml of GM-CSF and 20 ng/ml of IL-4, followed by plating 0.5 ml of culture (1X10^6^ cells/well) in a 12-well plate. On days 5 and 6, 250 µl of supernatant was replaced with concentrated lentivirus-containing media. On day 8, the cells were harvested, washed with PBS, and used for the experiment. For the control, a ten-fold concentrated complete medium with polybrene was utilized.

### Plasmid transfection

HEK293t cells (2.5 × 10^5^/well) were plated in a 12-well plate with 0.5 ml of complete DMEM medium for the cytokine assay. Additionally, 0.5 × 10^6^ cells/well were plated in a 6-well plate with 1 ml of medium for the immunoprecipitation assay. After 12 hours, the cells were transfected with constructs expressing NLRC4-WT-FLAG, NLRC4-WT-GFP, NLRC4^CARD(D25K)^-GFP, NLRC4 ^CARD(R52E)^-GFP, NLRC4^CARD (D25K/R52E)^- GFP, ASC-HA, CARD (ASC)-HA, Pro-caspase-1-His, Pro-IL-1β, Pro-IL-18, LRRK2-Myc, G2019S-GFP, D2017A-mCherry, and NAIP-FLAG, as indicated. The total DNA amount was adjusted to ensure equal concentration per well using an empty vector. Plasmid DNA was first diluted in Opti-MEM and then mixed with Lipofectamine LTX according to the manufacturer’s instructions (Thermo Fisher Scientific). This mixture was incubated at room temperature for 10 minutes and subsequently added to HEK293t cells with either 0.5 ml or 1 ml of complete DMEM culture medium. The following day, the medium was replaced with fresh DMEM, and cells were treated with or without Needle-tox (recombinant LFn-Needle antigen at 1 µg/mL, with recombinant anthrax protective (PA) antigen at 1 µg/mL) for three hours. The cell culture supernatant was collected for ELISA analysis. For interaction studies, 18 hours post-transfection, cells were harvested, lysed with NP-40 lysis buffer (detailed below), and subjected to immunoprecipitation (IP) and Western blot (WB) analysis. For cytokine level assays, plasmid concentrations were as follows: NLRC4: 250 ng; ASC: 100 ng; Pro-IL-1β: 50 ng; Pro-IL-18: 50 ng; LRRK2: 500 ng; G2019S: 500, D2017A: 500ng, NAIP: 100 ng. For immunoprecipitation assays, plasmid amounts were: NLRC4: 1000 ng; LRRK2: 1500 ng; ASC: 200 ng; CARD (ASC)-HA: 200 ng; Pro-caspase-1-His: 200 ng. Plasmid details are provided in **Supplementary Table 4**.

### Immunoblotting and immunoprecipitation

Cells to be subjected to Western blot (WB) or immunoprecipitation (IP) were lysed with lysis buffer (75 µl per well in a 6- well plate for 1 × 10^6^ cells or 35 µl per well in a 12- well plate for 0. 5 × 10^6^ cells) containing 50 mM Tris (pH 7.5. 5), 0. 0.5% NP- 40, 50 mM NaCl, 1 mM NaVO 4, 1 mM NaF, and a protease- and phosphatase- inhibitor cocktail. The cell lysates were kept on ice for 15 minutes and then pelleted by centrifugation at 13, 000 × g for 15 minutes. The supernatants were collected, and protein concentration was measured using a Nanodrop. An appropriate amount of protein was mixed with LDS loading buffer, heated at 70°C for 10 minutes, and run on LDS electrophoresis. Proteins were transferred onto nitrocellulose membranes (Bio- Rad, San Diego, CA), blocked with 5% fat- free milk in 1 × TBS with 0. 05% Tween 20, and probed with primary antibodies specific to the target proteins. After overnight incubation at 4°C, the primary antibodies were removed, and the membranes were washed three times for five minutes each with 1 × TBS containing 0. 05% Tween- 20. They were then incubated with appropriate horseradish peroxidase- conjugated secondary antibodies at room temperature for one hour. The membranes were washed five times at fifteen- minute intervals with 1 × TBS with 0. 05% Tween- 20. Immunoreactive bands were visualized using an enhanced chemiluminescence reagent (K- 12043- D 10, Advansta). Immunoprecipitation was performed using the DynabeadsTM Protein G Immunoprecipitation Kit (Thermo Fisher Scientific, 10007 D). Ten microliters of dynabeads per IP sample were washed in PBS, resuspended in 250 µl of lysis buffer, and mixed with 1 µg of antibody per IP sample. The beads- antibody mixture was incubated at room temperature for 30 minutes. After incubation, the prepared cell lysates were added to the antibody containing beads. The beads- antibody- cell lysate mixtures were incubated on a rotator overnight at 4°C, then washed with lysis buffer three times for five minutes each, and subjected to Western blot analysis.

### ASC oligomerization

ASC oligomerization detection was performed following the methods previously described by Mao et al. (13). Briefly, 1×10^6^ dendritic cells per well in a 12-well plate were stimulated as indicated, with three wells (3×10^6^ cells) used for each stimulation condition. After stimulation, cells were lysed with 250 μl of lysis buffer as described earlier. The cell lysates were then passed through a 21-gauge needle 10 times and incubated on ice for 15 minutes. Subsequently, the lysates were centrifuged at 13,000g for 15 minutes at 4°C. The supernatants were collected, and insoluble cell debris was resuspended in 0.5 ml of PBS and crosslinked with freshly prepared disuccinimidyl suberate (DSS; S1885, Sigma-Aldrich) at a concentration of 0.8 mM for 30 minutes at 37°C. Finally, the crosslinked debris was pelleted by centrifugation at 6,000g for 10 minutes and resuspended in 30 μl LDS loading buffer for Western blot detection of ASC.

### ASC speck quantitation

PBMC-derived dendritic cells (PB-DCs), generated over 7 days as described in Methods and adjusted to a concentration of 1×10^6^ cells/mL, were placed in an ibidi 8-well slide chamber at 200 µl (2×10^^5^ cells per well). The following morning the culture media were replaced with fresh media containing 0.1% DMSO (vehicle control) or LRRK2 inhibitors, LRRK2-IN-1 or CZC 54252.HCl (5 µM for 60 minutes) and cultured with Needle-Tox (1 µg/mL, 3 hours) following which the cells were fixed with 4% paraformaldehyde for 15 minutes at room temperature and washed with PBS twice for 5 minutes each. At this point the cells were treated with 0.1% Triton X-100 for 20 minutes at room temperature, followed by two washes with PBS for 5 minutes each. Then, after blocking with 2% BSA (Bovine Serum Albumin, Sigma, USA) at room temperature for 30 minutes, the cells were incubated with anti-ASC antibody (1:500) (Novus Biologicals, USA) overnight at 4°C. after which they were washed three times for 15 minutes with PBS, incubated with secondary antibody (anti-mouse IgG, Thermo Fisher Scientific, USA) at room temperature for 1 hour, washed three times for 10 minutes with PBS and incubated with DAPI for 5 minutes at room temperature. Finally, the cells were washed with PBS and examined under a confocal microscope to enable counting of specks.

### Mouse infection and permeability measurement

Mice received LPS (0.1 mg/kg) from Day 0 to Day 3 and were co-injected with LFn-flagellin (0.04 µg/g) and PA (0.08 µg/g) from Day 1 to Day 3. Where applicable, LRRK2 inhibitors were administered at 5 mg/kg from Day 1 to Day 3. On Day 4, mice fasted for 1 hour, then 150 µl of FD4 (Sigma-Aldrich, USA) solution (10 mg/mL) was orally given. One hour after gavage, mice were euthanized, and blood samples collected. Blood was left at room temperature for 2 hours, then centrifuged at 8,000 g for 7 minutes at 4°C to isolate plasma. FD4 fluorescence was measured in duplicate using a spectrophotometer (SpectraMax M4, Molecular Devices, San Jose, CA, USA) with excitation at 485 nm and emission at 535 nm.

### Intestinal tissue lysate

Whole intestines from mice were collected and homogenized using a tissue homogenizer (Tissue Master 125, Omni International, USA) in 1.5 ml of cold PBS. The homogenates were then centrifuged at 14,000 rpm for 30 minutes at 4°C. The supernatants were carefully collected, and the pellets were discarded. This process was repeated three additional times to ensure clarity. Finally, the clear supernatants were stored at -80°C until ELISA analysis was performed.

### ELISA

Supernatants from cell cultures or whole intestinal lysates were collected and used for ELISA samples. The mouse TNFα ELISA kit was obtained from BD Bioscience, and the mouse IL-1β and IL-18 kits are from R&D. The ELISA was performed according to the manufacturer’s instructions.

### Statistical analysis

GraphPad Prism version 10.3.1 (GraphPad Software, San Diego, CA, USA) was used for statistical analysis; p-values below 0.05 were considered significant. Group comparisons were made using a parametric Student t-test. Image Lab software (Bio-Rad) was used for image analysis.

## Data Availability

The original contributions presented in the study are included in the article/**Supplementary Material**. Further inquiries can be directed to the corresponding authors.
