# Supplementary material for: LRRK2-mediated NLRC4 phosphorylation differentially regulates IL-1β/IL-18 secretion
Source: Front Immunol. 2025 Oct 30;16:1675137. doi: 10.3389/fimmu.2025.1675137 (PMC12611935; doi:10.3389/fimmu.2025.1675137)

LRRK2-Mediated NLRC4 Phosphorylation Differentially Regulates IL-1β/IL-18 Secretion

Supplementary Figure 1:

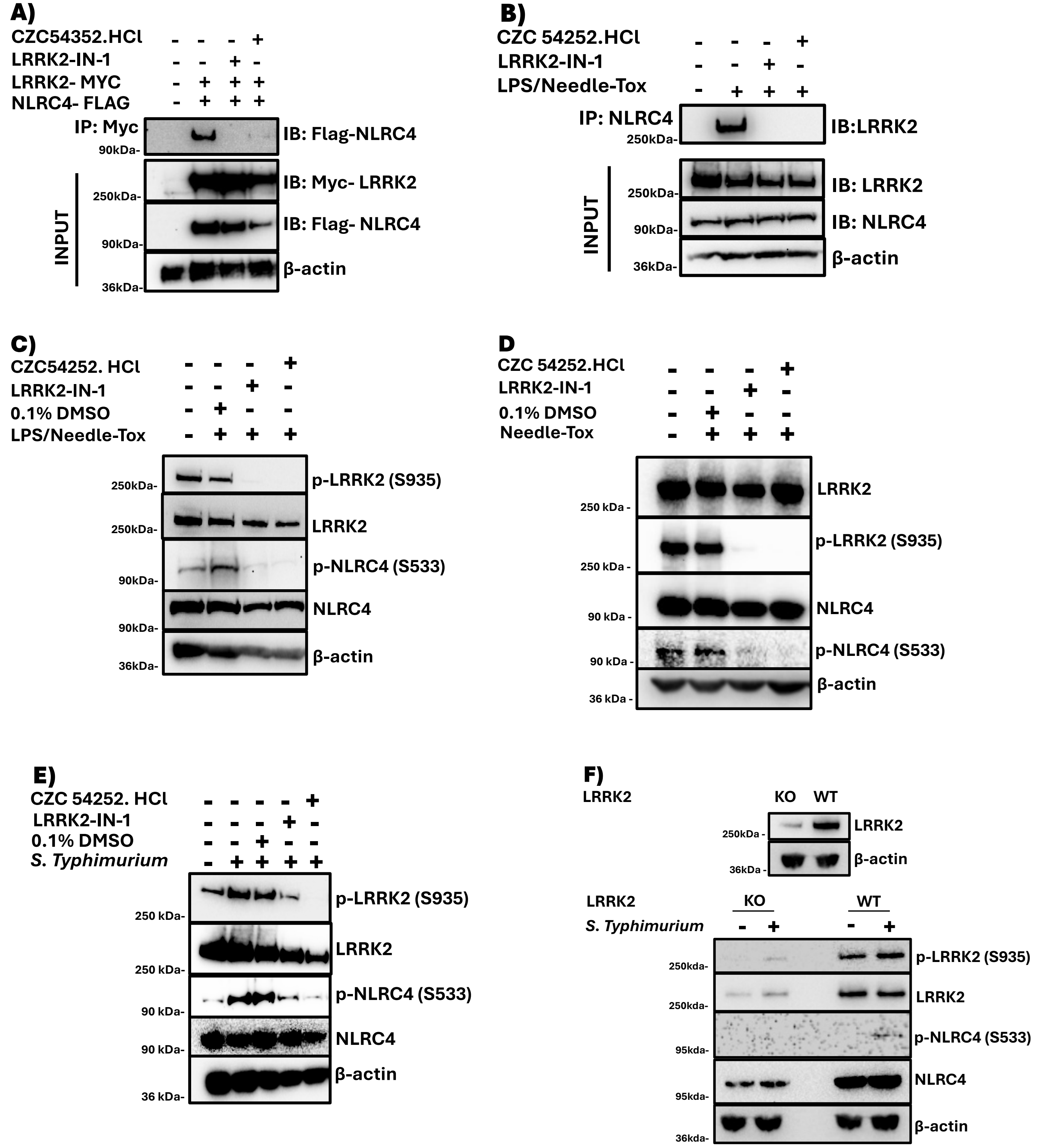

Supplementary Figure 1: LRRK2 Kinase Inhibitors Block LRRK2/NLRC4 Interaction and NLRC4 Phosphorylation at Ser53

(A) The HEK 293t cells were transfected with plasmids expressing MYC-tagged LRRK2 and co-transfected with FLAG-tagged NLRC4; after 24 hours, the media was replaced with fresh complete DMEM containing LRRK2 inhibitors LRRK2-IN-1 and CZC 54252.HCl at 5 μM, then treated for 2 hours. Finally, the cells were lysed, and the lysates were subjected to immunoprecipitation with anti-MYC antibodies, followed by immunoblot analysis with the indicated antibodies. (B) PBMC-derived dendritic cells (PBDCs), generated over 7 days as described in Methods and adjusted to a concentration of $1x10^6$ cells/ml, were placed in a 12-well culture plate at 1 ml per well. The next morning, the media was removed and replaced with fresh media containing either 0.1% DMSO alone or one of two LRRK2 inhibitors, LRRK2-IN-1 and CZC 54252.HCl at 5 μM for 60 minutes. Subsequently, they were stimulated with LPS (200 ng/ ml, 3 hours) followed by Needle-Tox stimulation (1 μg/ml, 30 minutes). Cells were lysed, and the lysates were immunoprecipitated with anti-NLRC4 antibodies, then analyzed by immunoblot. **(**C) PB-DC lysates from (B) were analyzed by immunoblot with the indicated antibodies. (D-E) PB-DCs treated as in (B) were stimulated with either needle-tox (1 μg/ml, 3 hours) or *S. typhimurium* (MOI 1, 4 hours), then lysed and analyzed by western blot with the indicated antibodies. (F) PBDCs underwent LRRK2 knockdown via CRISPR-Cas9-mediated gRNA insertion as described in Methods; they were then stimulated with *S. typhimurium* (MOI 1, 4 hours). Afterward, the cells were lysed, and the lysates subjected to immunoblot

analysis with the indicated antibodies. All results are representative of at least two independent experiments.

Supplementary Figure 2:

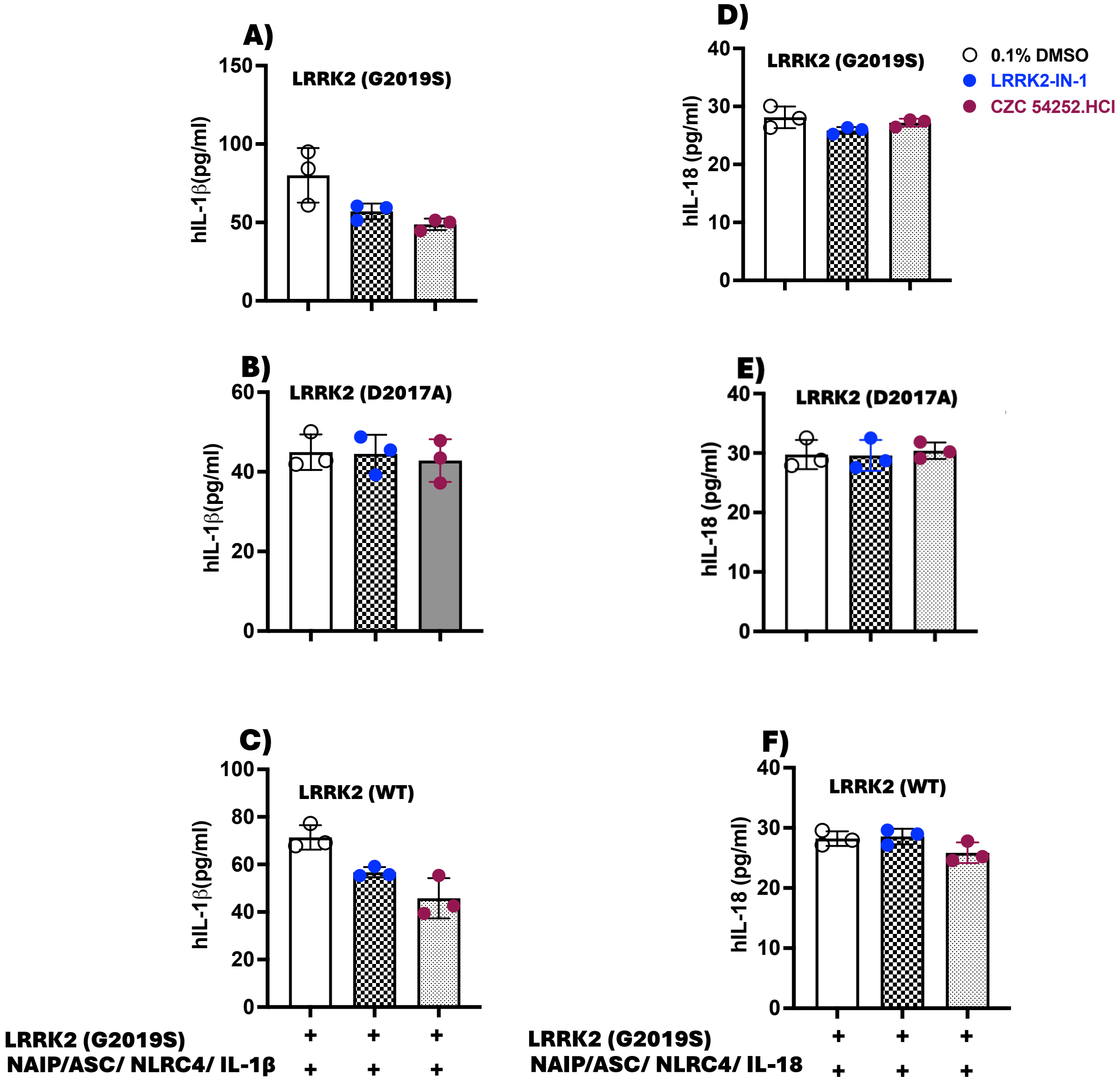

Supplement:

Supplementary Figure 2: LRRK2 Kinase Inhibitors Block IL-1β but not IL-18 Production

HEK293t cells were transfected with plasmids expressing either G2019S-LRRK2 (A and D), or dead kinase D2017A-LRRK2 (B and E), or wild-type LRRK2 (C and F) together with NLRC4, NAIP, ASC, and either IL- 1β (A-C) or IL-18 (D-F); after 6h cells were treated with LRRK2 inhibitors (LRRK2-IN-1 and CZC 54252.HCl, 5 μM) for 16h, after which cell culture supernatant was collected and IL-1β and IL-18 were measured by ELISA.

Supplementary Figure 3:

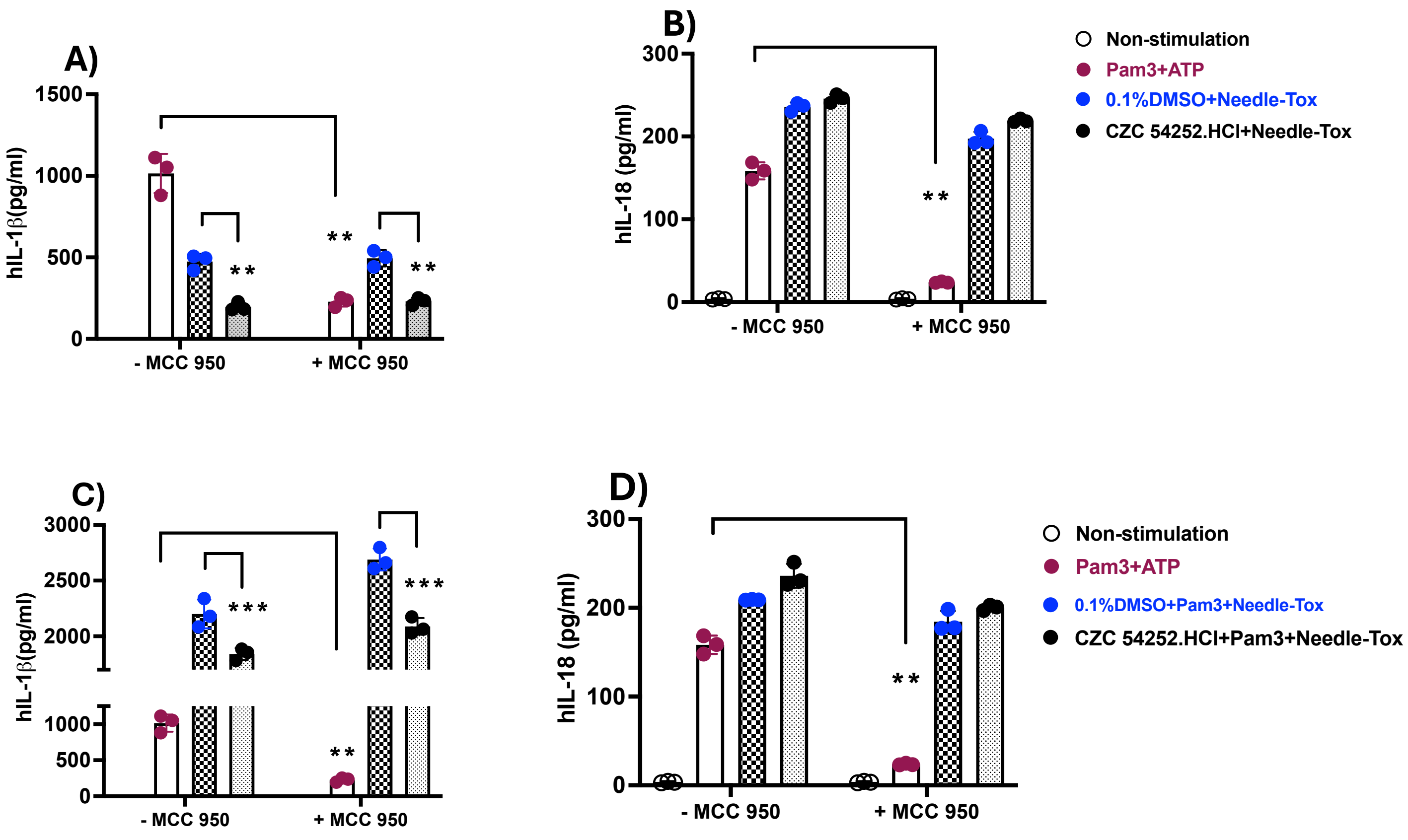

Supplementary Figure 3: LRRK2 Kinase Inhibitors act independently of NLRP3 suppression

(A-D) PBMC-derived dendritic cells (PBDCs), generated over 7 days as described in Methods and adjusted to a concentration of $1x10^6$ cells/ ml, were placed in a 12-well culture plate at 1 ml per well. The following morning, the culture media were replaced with fresh media containing 0.1% DMSO (vehicle control) or the NLRP3 inhibitor MCC950 (3 µM) for 30 minutes. Cells were then stimulated with Pam3CSK4 (100 ng/ ml, 3 hours) and followed by ATP (5 mM, 30 minutes). In parallel, after MCC 950 pre-treatment, cells were incubated with LRRK2 inhibitors, LRRK2-IN-1 or CZC 54252.HCl, (5 µM for 60 minutes), then stimulated with either Needle-Tox alone (1 µg/ ml, 4 hours) or Pam3CSK4 (100 ng/ ml, 3 hours), followed by Needle-Tox stimulation (1 µg/ ml, 30 minutes). Cell culture supernatants were collected for cytokine measurement by ELISA.

Supplementary Figure 4:

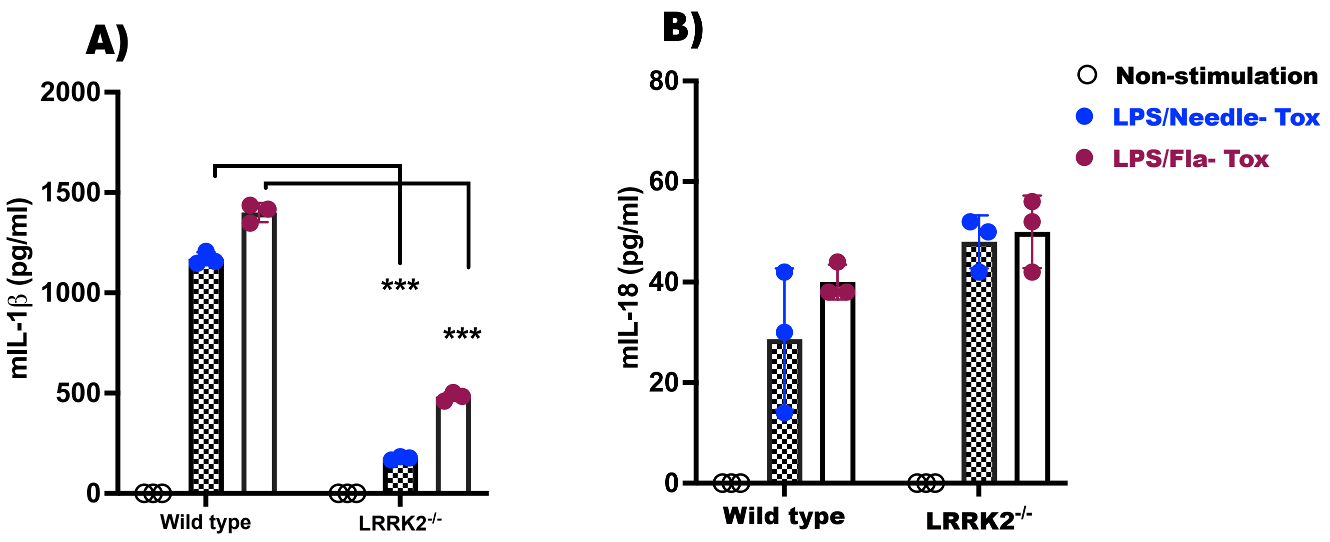

Supplementary Figure 4: NLRC4 Phosphorylation in Murine BMDCs is Dependent on LRRK2-Kinase and Affects NLRC4 Inflammasome IL-1β but not IL-18 Production

Mouse bone marrow-derived dendritic cells (BMDCs), generated and cultured as in Figure 2, were exposed to LPS (200 ng/ml) for 3 hours, followed by treatment with either Fla-Tox (1 μg/ml) for 1 hour or needle tox (1 μg/ml) for 30 minutes. (A-B) Cell culture supernatants were collected and analyzed for IL-1β and IL-18 levels using ELISA; cytokine levels shown are adjusted by subtracting the levels in unstimulated cultures. Data are presented as means ± SEM; *, $P < 0.05$; **, $P < 0.01$; ***, $P < 0.0001$; determined using Student's t-test. All conditions were tested in triplicate. In each panel, the data represent at least two independent experiments.

Supplementary Figure 5:

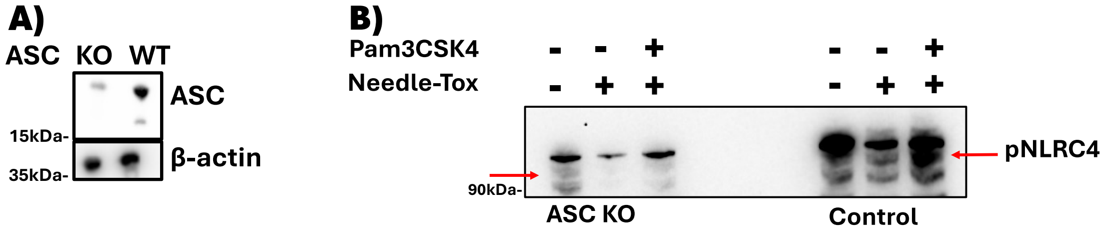

Supplementary Figure 5: NLRC4 phosphorylation requires ASC incorporation into the NLRC4 Inflammasome

PBDCs were subjected to ASC knock-out (KO) via CRISPR-Cas9 mediated gRNA insertion as described in Methods; (A) immunoblot of WT and ASC KD PB-DCs, (B) WT (control) and partially ASC KO PBDCs were treated with or without Pam3CSK4 (100 ng/ml, 3h), and subsequently with or without Needle-Tox (1 μg/ml, 30 min or 3h) as indicated after which the cells were lysed and the lysates obtained were subjected to immunoblot analysis with the indicated antibodies.

Supplementary Figure 6:

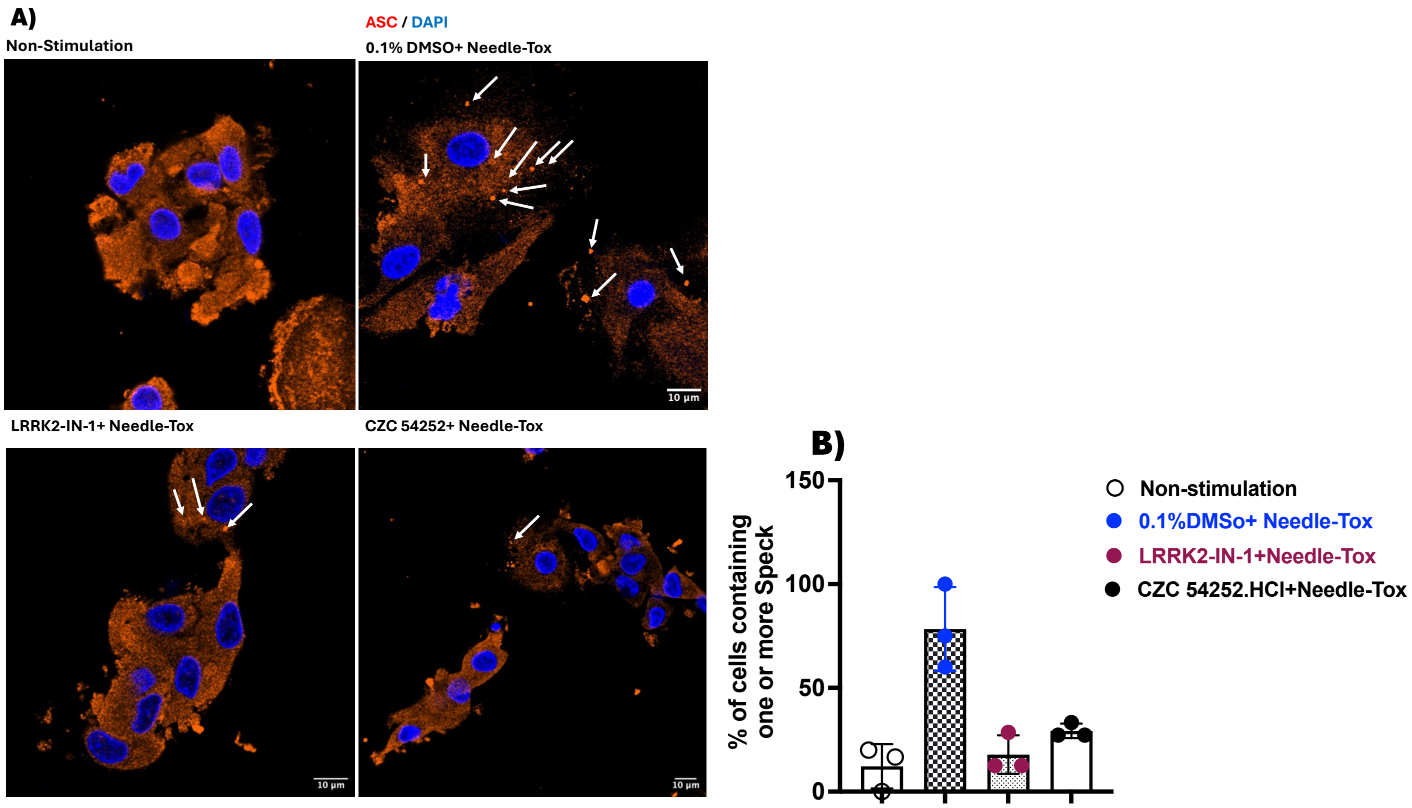

Supplementary Figure 6: LRRK2 kinase inhibitors inhibit ASC-speck formation.

PBMC-derived dendritic cells (PBDCs), generated over 7 days as described in Methods and adjusted to a concentration of $1×10^6$ cells/ml, were placed in an ibidi 8-well slide chamber at 200 µL per well. The following morning, the culture media were replaced with fresh media containing 0.1% DMSO (vehicle control) or LRRK2 inhibitors, LRRK2-IN-1 or CZC 54252.HCl (5 µM for 60 minutes). Then, the cells were stimulated with Needle-tox (1 µg/ ml, 3 hours). ASC speck formation was assayed by ASC

immunofluorescent staining, and cells were counterstained with DAPI (blue) as indicated in Methods. Finally, fluorescent images were analyzed using confocal microscopy, and the images were processed with ImageJ Fiji.

Supplementary Figure 7:

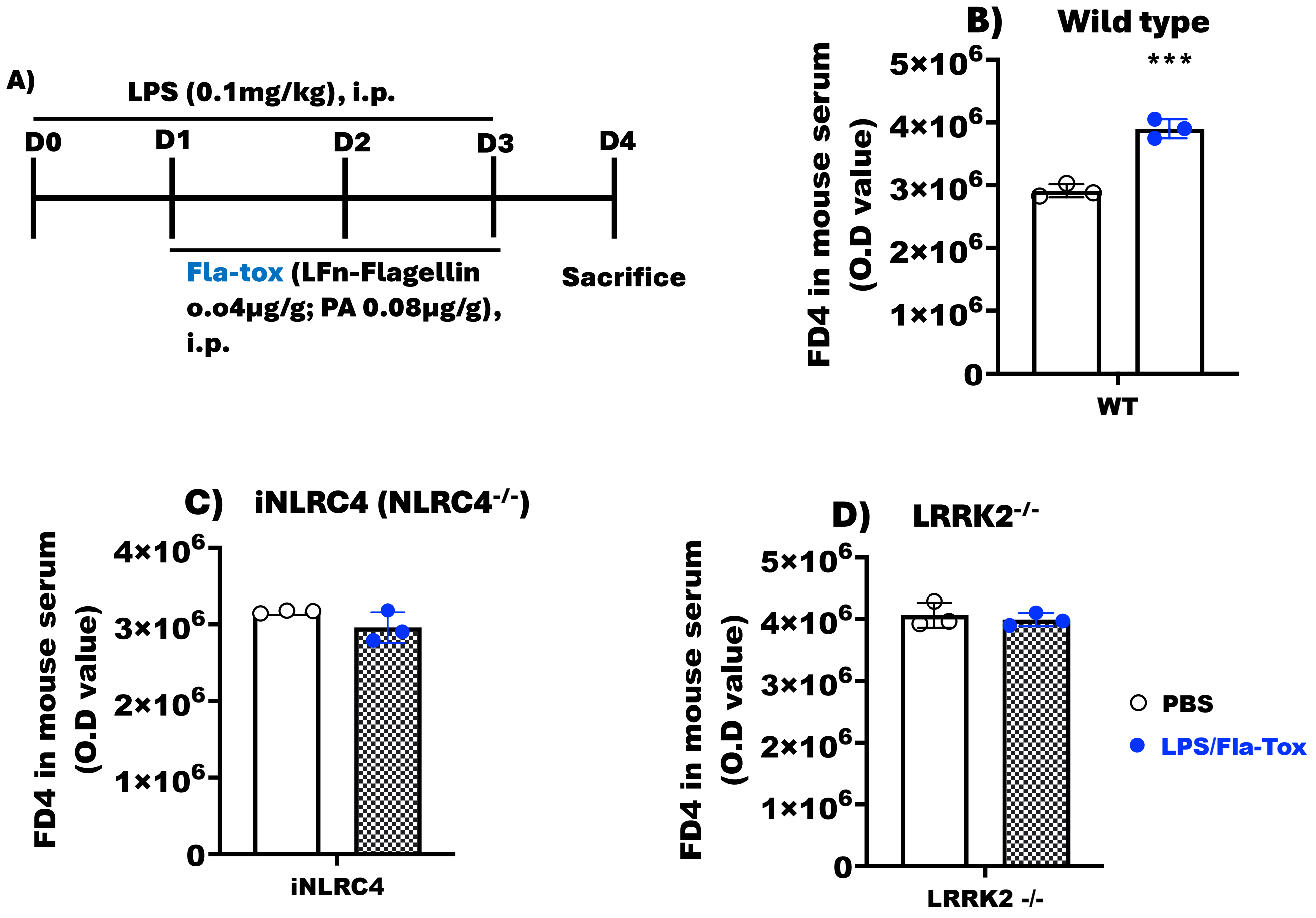

Supplementary Figure 7: Intestinal Permeability Induced by the NLRC4 Inflammasome Activation

(A) As per the illustrated protocol, 8-10 week old C57BL/6 (Jackson, Strain# 000664) WT mice, in-house iNLRC4 (NLRC4 KO) mice, and LRRK2 KO mice (n=3 per group) were administered PBS alone or PBS containing LPS (0.1 mg/kg) (IP) daily for 4 days, as well as Fla-Tox (LFn-Flagellin, 0.04 μg/g + PA 0.08 μg/g) (IP) for 3 days. On day 4, FITC dextran (FD4) (10 mg/ml, 150 μl/mouse) was administered (PO), and blood was collected at the time of euthanasia. (B-D) OD values of FD4 measured in blood serum as described in Methods. Each dot indicates one mouse. Data are shown as means ± SEM; *, $P < 0.05$; **, $P < 0.01$; ***, $P < 0.0001$, determined by Student's t-test. All conditions were tested in triplicate. In each panel, data represent at least two independent experiments.

Supplementary Table I: Crohn’s Disease patients' (Figure 5A – B) disease status

Barcode	Age	Gender	Lab values
1098415	37	F	ESR=23, CRP=1.1
1098532	31	F	no labs
1099640	35	M	Calpro 183, CRP 1.2
1098503	29	F	no labs
1100838	27	F	ESR=13, CRP 2.4
1100870	39	F	no labs
1099105	48	F	ESR=40(H), CRP =1.5
1097419	40	M	CRP= 2.5
1097423	41	M	CRP=9.9 (H), ESR=37 (H)
1097569	35	M	CRP=7.8, ESR= 24
1100466	35	F	CRP=28.5, ESR=42 (H)
1100928	35	F	CRP=3, ESR=91 (H)
1100929	63	F	CRP=0.5, ESR=20
1100982	52	F	CRP=16.5 (H)
1100983	37	F	CRP=0.5, ESR=20
1101011	69	F	CRP=1.6, ESR=15

Supplementary Table II: Crohn's Disease patients' (Figure 5C – D) disease status

Barcode	Age	Gender	Lab values
1109529	40	M	Calpro 28, CRP <3, ESR 9
1109535	35	M	CRP <3, ESR 14
1109537	32	M	Calpro 964 (H), CRP <3, ESR 52 (H),
1109556	30	F	CRP <3, ESR 14
1109601	27	M	CRP <3
1109779	42	F	Calpro 11, CRP 27.2 (H)
1109852	35	M	CRP <3, ESR 25
1109853	39	F	CRP 9.1 (H) better than 3 months ago
1109854	38	M	CRP <3, ESR 23
1110188	39	M	CRP <3, ESR 7
1110208	63	F	CRP <3, ESR 41 (H)
1110298	70	F	CRP <3, ESR 20
1110984	40	M	CRP <3, ESR 21
1110961	62	M	CRP <4.1, ESR 36

Supplementary Table III: List of antibodies

Antibody	Cat#	Source
Rabbit anti-LRRK2 (MJFF3 (c41-2)	ab133474	Abcam
anti-phospho-LRRK2 (S935) (UDD2 10[15])	ab 133450	Abcam
Rabbit anti-IPAF (NLRC4	06-1125	EMD Millipore
Rabbit anti-NLRC4 (D5Y8E)	12421	Cell Signaling Technology
Rabbit polyclonal anti-phospho NLRC4	NP 5411	ECM Biosciences
Mouse anti Pro-IL-1b (3A6)	12242	Cell Signaling Technology
Rabbit anti Cleaved-IL-1b (Asp 116, D3A3Z)	83186	Cell Signaling Technology
Rabbit anti IL-18 (D2F3B)	54943	Cell Signaling Technology
Rabbit anti IL-18 (EPR 19956)	ab 207323	Abcam
Caspase-1/p20/p10	22915-1-AP	Proteintech
Rabbit anti ASC (E1E31)	13833	Cell Signaling Technology
Anti MYC (71D10)	2278	Cell Signaling Technology
Anti HA ((C29F4)	3724	Cell Signaling Technology
Anti His	2365	Cell Signaling Technology
Anti FLAG (D6W5B)	14793	Cell Signaling Technology
Rabbit anti-β-actin (D6A8)	8457	Cell Signaling Technology
Anti Rabbit IgG	7074	Cell Signaling Technology
Anti mouse IgG	7076	Cell Signaling Technology
Anti ASC/TMS1(1C3D7)	NBP2-61682	Novus Biologicals
Anti-mouse IgG (H+L), $F(ab')_2$ Fragment (Alexa Fluor® 594 Conjugate)	#8890	Cell Signaling Technology
DAPI	#D1306	Thermofisher Scientific

Supplementary Table IV: List of reagents

Inhibitors and Recombinant antigen	Cat#	Source
LRRK2-IN-1	4273	Tocris
CZC 54252.HCl	4534	Tocris
	1191911-27-9	Adooq bioscieces
MCC 950	A gift from Dr. Lucena Lage Silvia, NIAID, NIH	
Recombinant LFn-needle	Ordered	LifeSct, LLC, USA
Recombinant LFn-flagellin	Ordered	LifeSct, LLC, USA
Anthrax protective antigen (PA 63)	cat# 174	Listlabs, CA, USA
LPS	tlrl-3pelps	InvivoGen, USA
Pam3CSK4	tlrl-pms	InvivoGen, USA
FD4 (Fluorescein isothiocyanate-dextran)	60842-46-8	Sigma-Aldrich
**Plasmid**		
NLRC4- FLAG	OHu 70975D	GenScript
NLRC4-WT- GFP	Customized order	LifeSct LLC
NLRC4$^{CARD (D25K)}$- GFP	Customized order	LifeSct LLC
NLRC4$^{CARD (R52E))}$- GFP	Customized order	LifeSct LLC
NLRC4$^{CARD (D25K/R52E)}$- GFP	Customized order	LifeSct LLC
LRRK2-MYC	#25361	addgene
LRRK2- G2019S-GFP	#25045<br>A gift from Francesco G. Barone, NINDS, NIH	addgene
LRRK2- D2017A-mCherry	A gift from Francesco G. Barone, NINDS, NIH	MRCPPUREAGENTS
ASC-HA	#41553	addgene
CARD (ASC)- HA	Customized order	LifeSct LLC
NAIP	Customized order	GenScript
IL-1 β	Customized order	GenScript
IL-18	Customized order	GenScript

Supplement:

Caspase-1-His	Customized order	GenScript
**ELISA kit**		
Human IL-1β ELISA kit	557953	BD Biosciences
Mouse IL-1β ELISA kit	DY401-05	R&D system
Human IL-18 ELISA kit	DY318-05	R&D system
Mouse IL-18 ELISA kit	DY7625-05	R&D system

Supplement:

Raw Images:
Figure 1:

Figure 1C

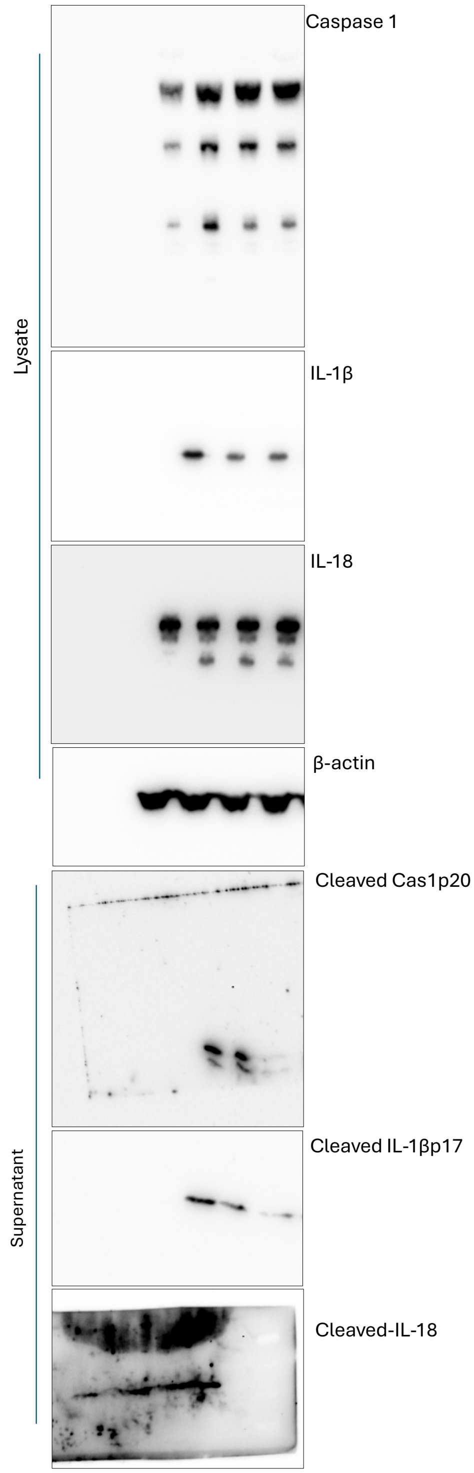

Figure 1F

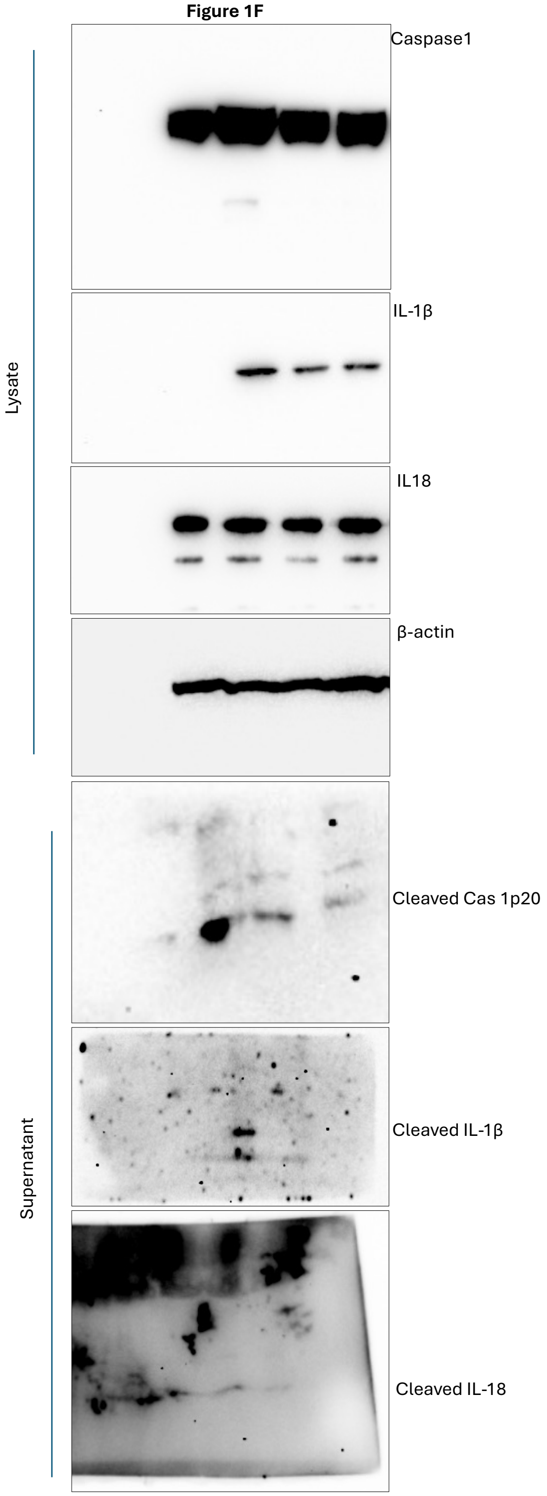

Supplement:

Figure 1I

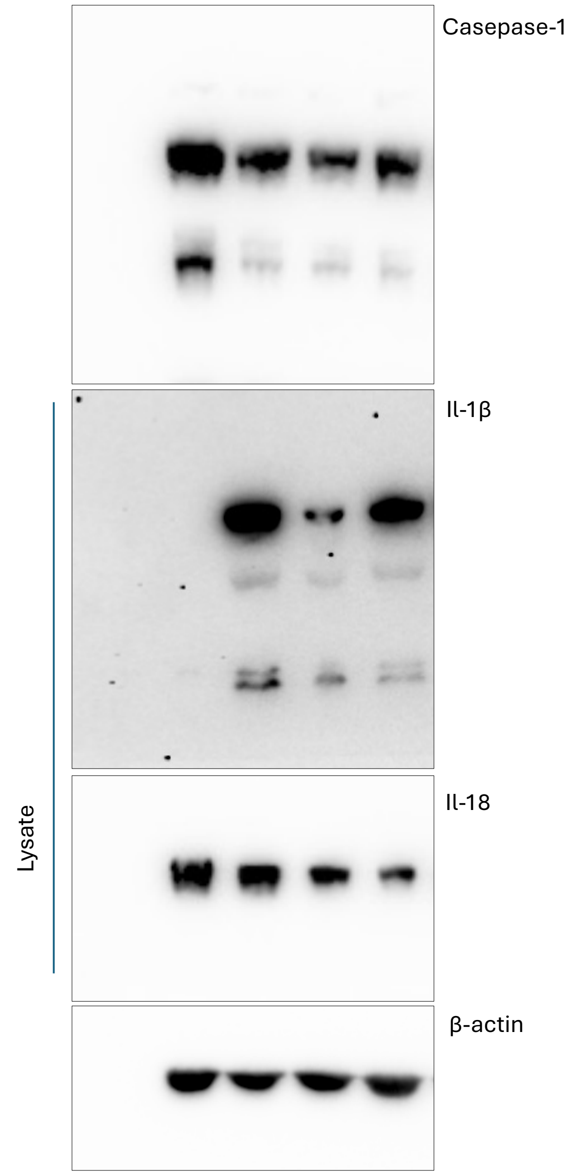

Figure 1L

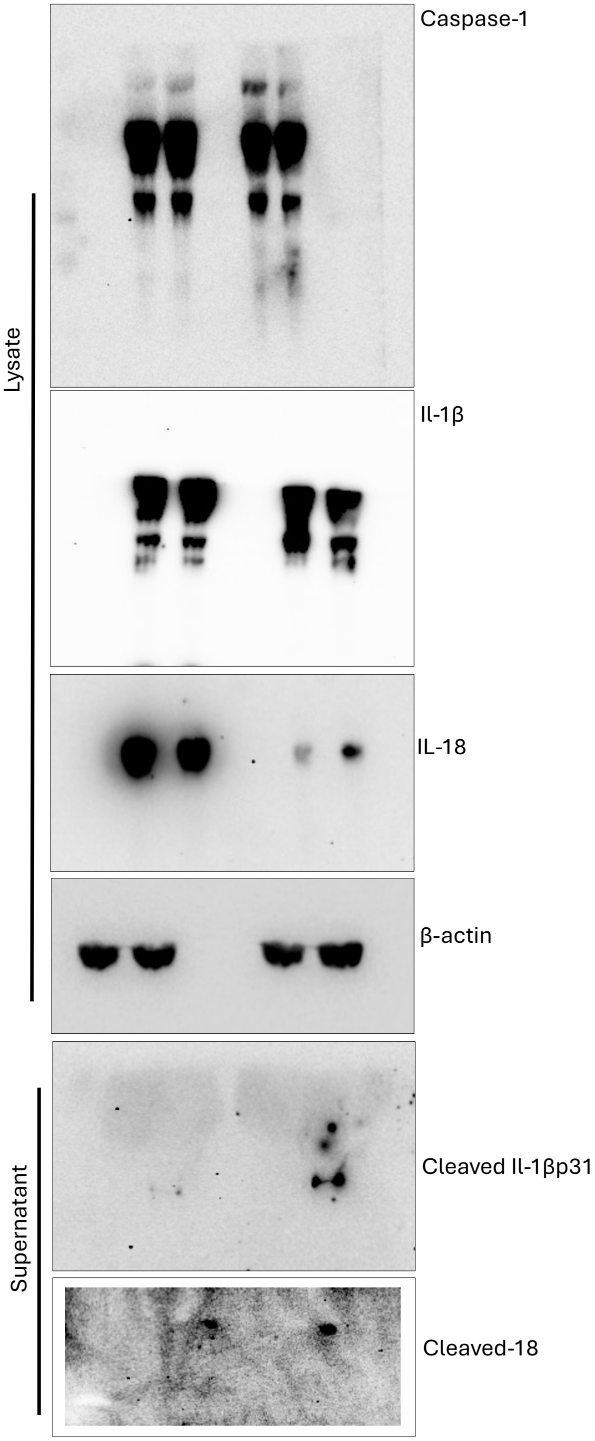

Supplement:

Figure 2:

Figure 2E

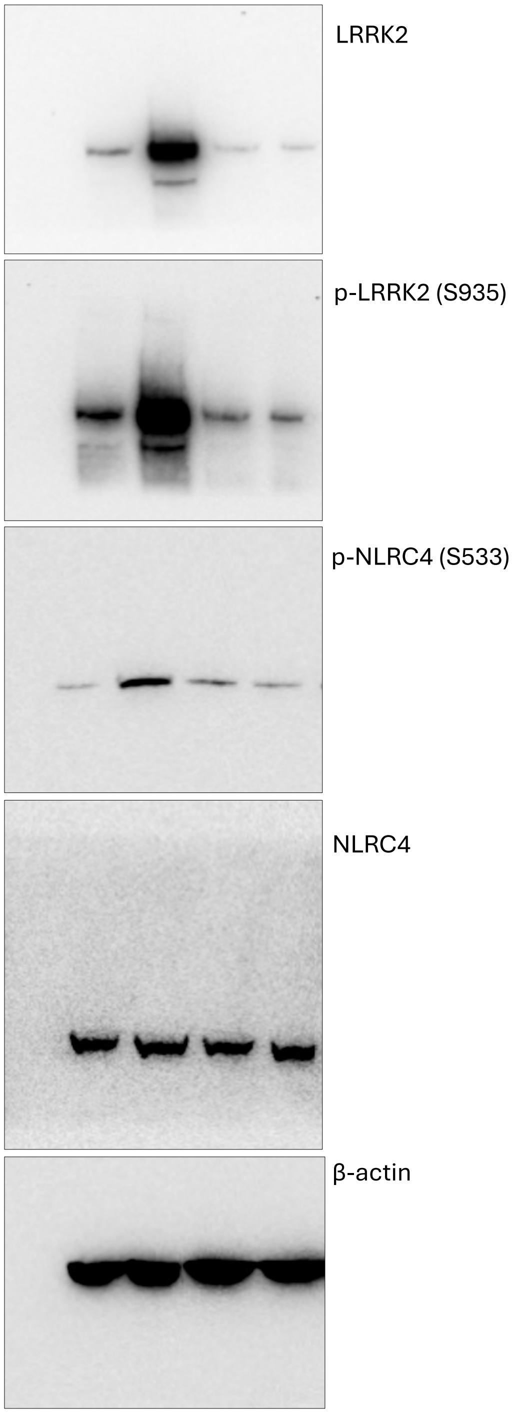

Figure 2F

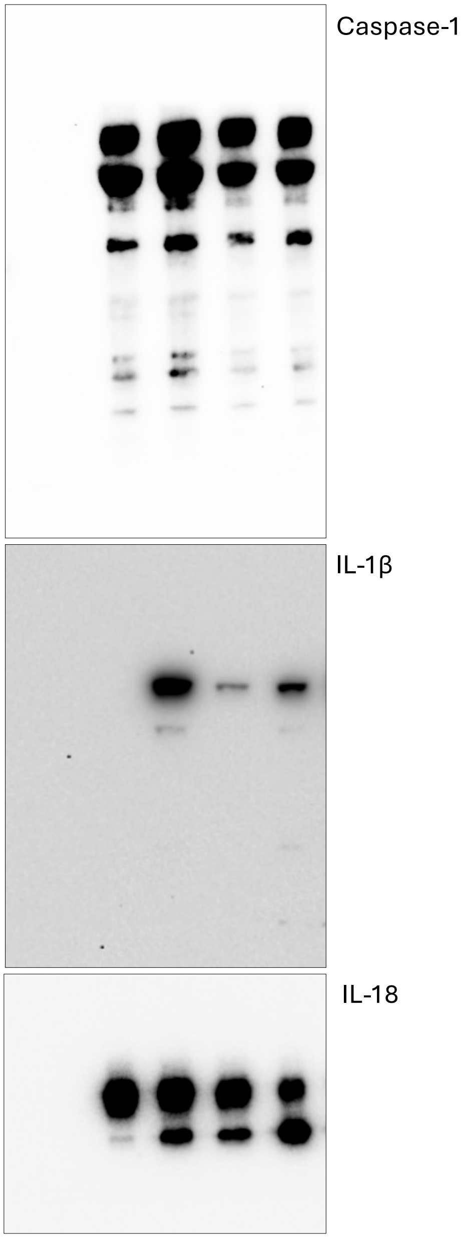

Supplement:

Figure 2G

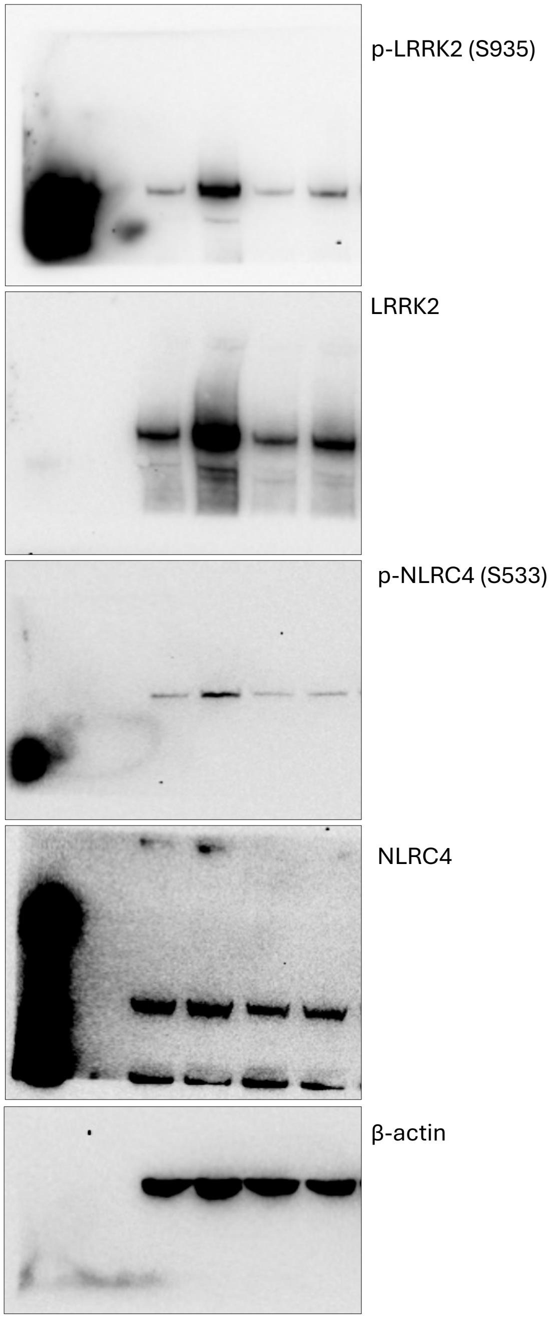

Figure 2H

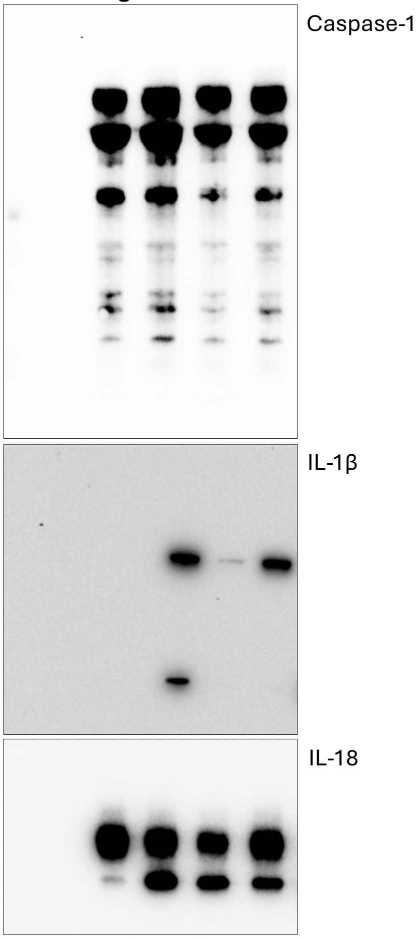

Supplement:

Figure 3:

Figure 3A

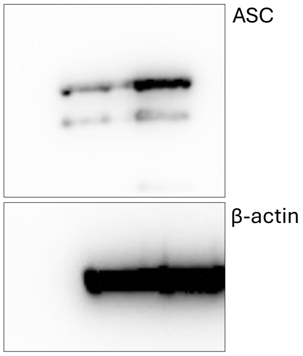

Figure 3B

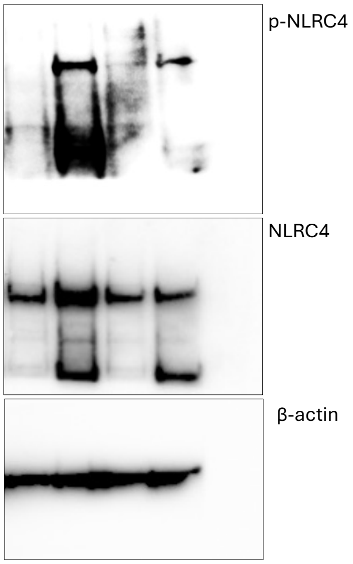

Figure 3C

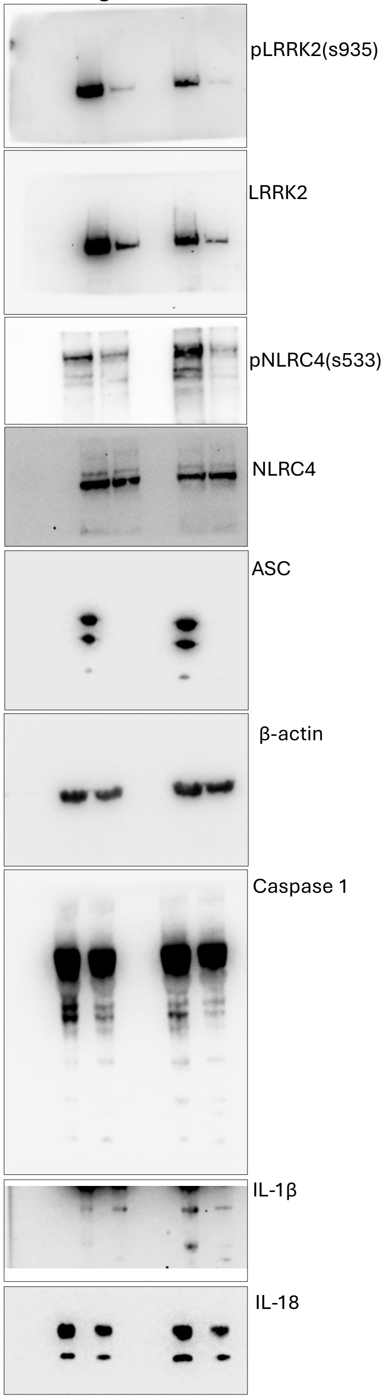

Supplement:

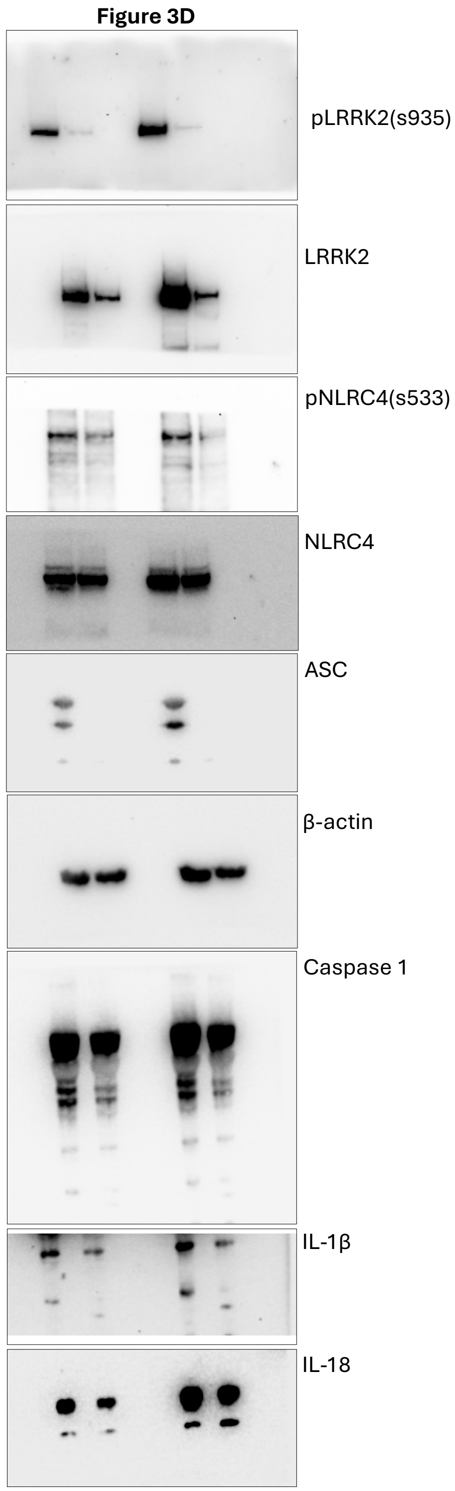
Figure 3D
pLRRK2(s935)
LRRK2
pNLRC4(s533)
NLRC4
ASC
β-actin
Caspase 1
IL-1β
IL-18

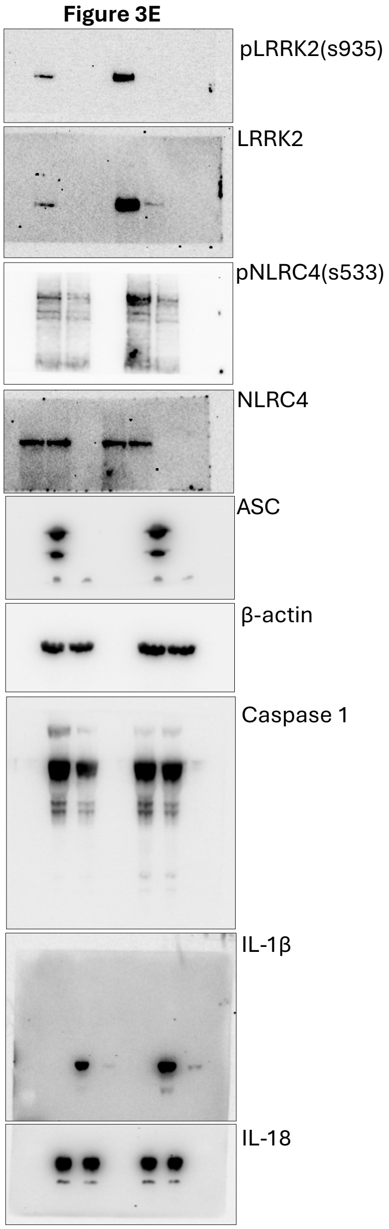
Figure 3E
pLRRK2(s935)
LRRK2
pNLRC4(s533)
NLRC4
ASC
β-actin
Caspase 1
IL-1β
IL-18

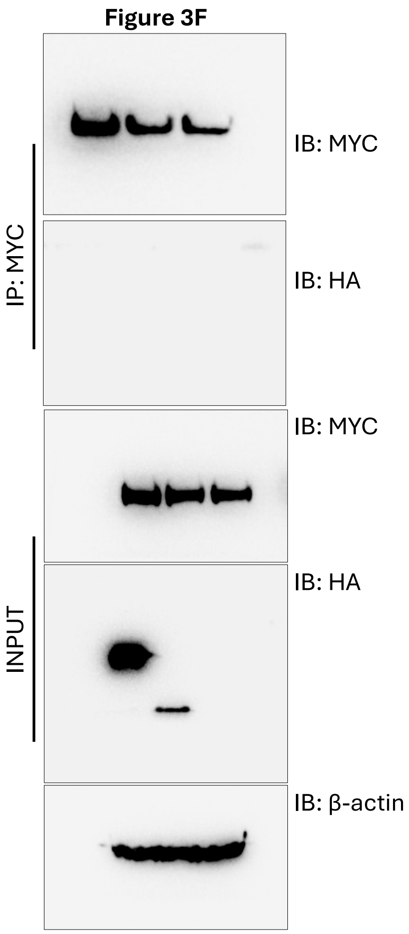
Figure 3F
IP: MYC
IB: MYC
IB: HA
INPUT
IB: MYC
IB: HA
IB: β-actin

Supplement:

Figure 4:

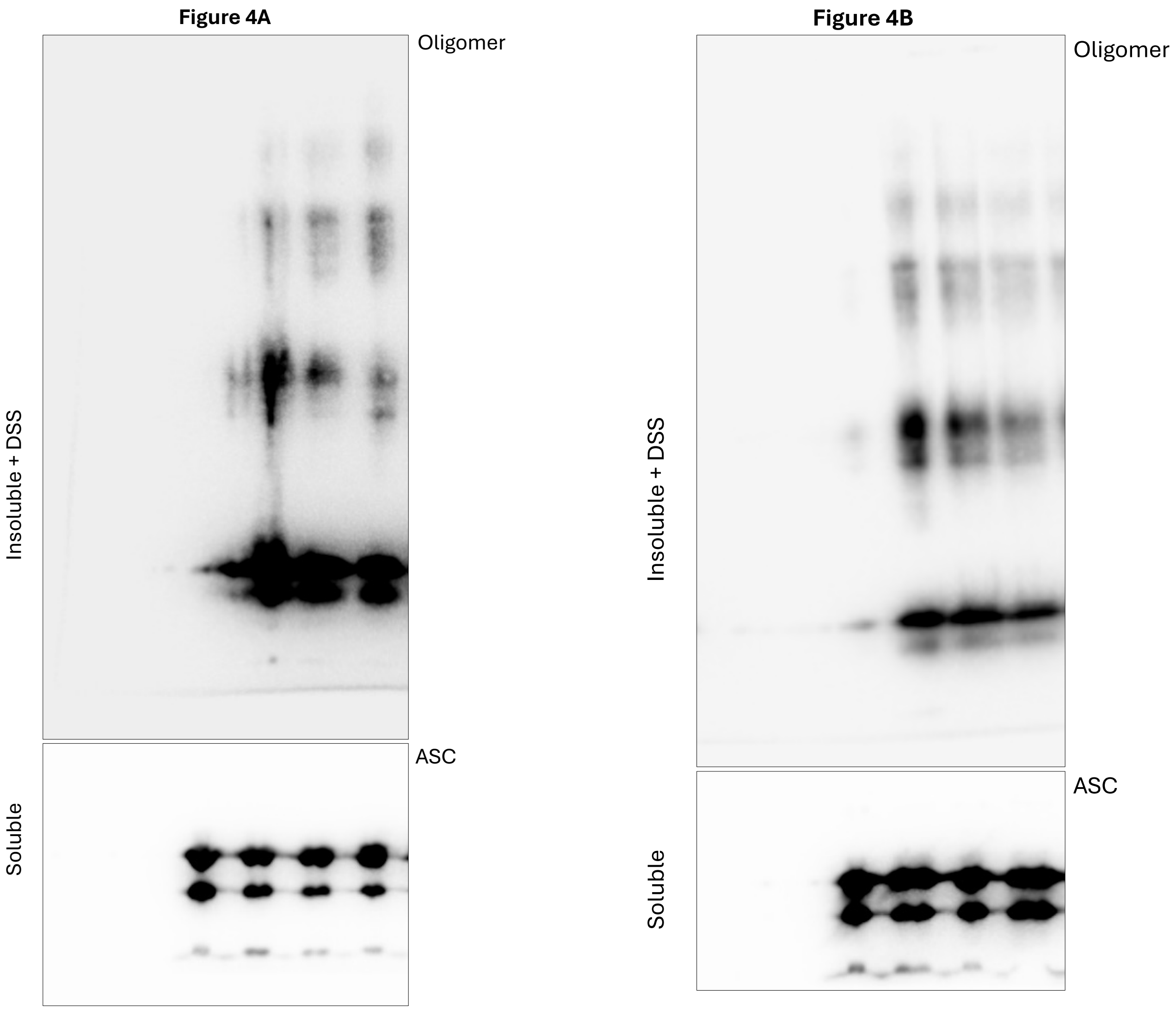

Supplement:

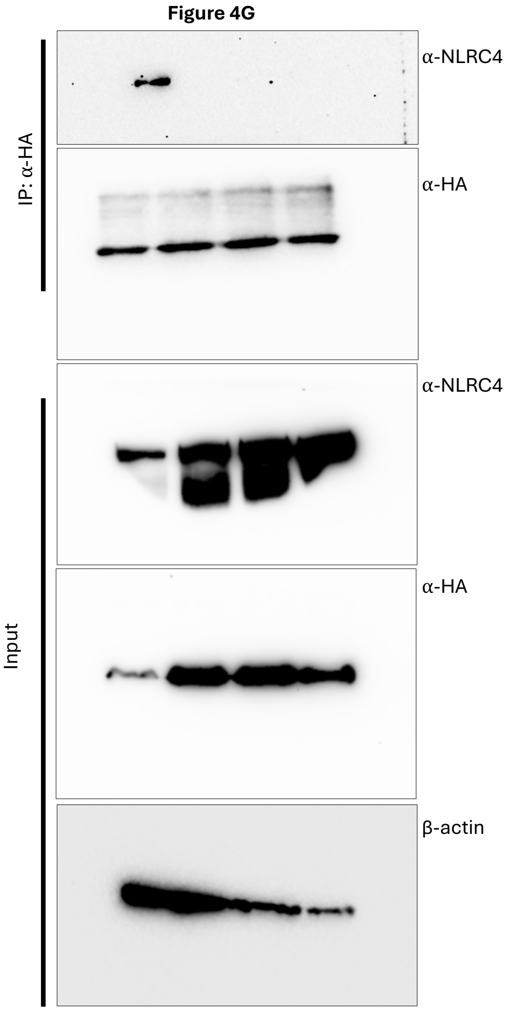
Figure 4G
α-NLRC4
α-HA
IP: α-HA
α-NLRC4
α-HA
β-actin
Input

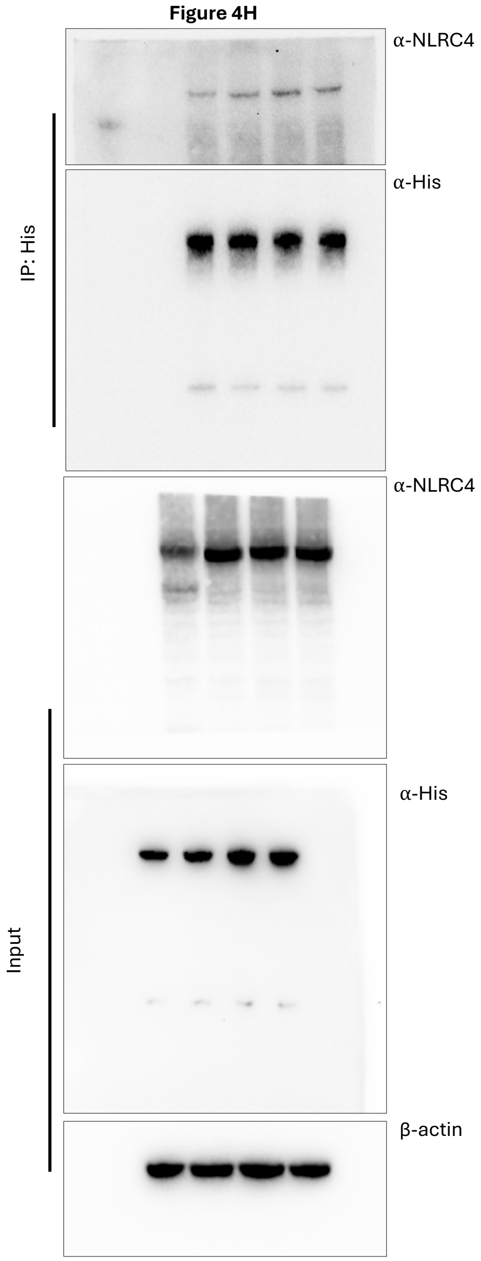
Figure 4H
α-NLRC4
α-His
IP: His
α-NLRC4
α-His
β-actin
Input

Supplementary:

Supplementary Figure 1:

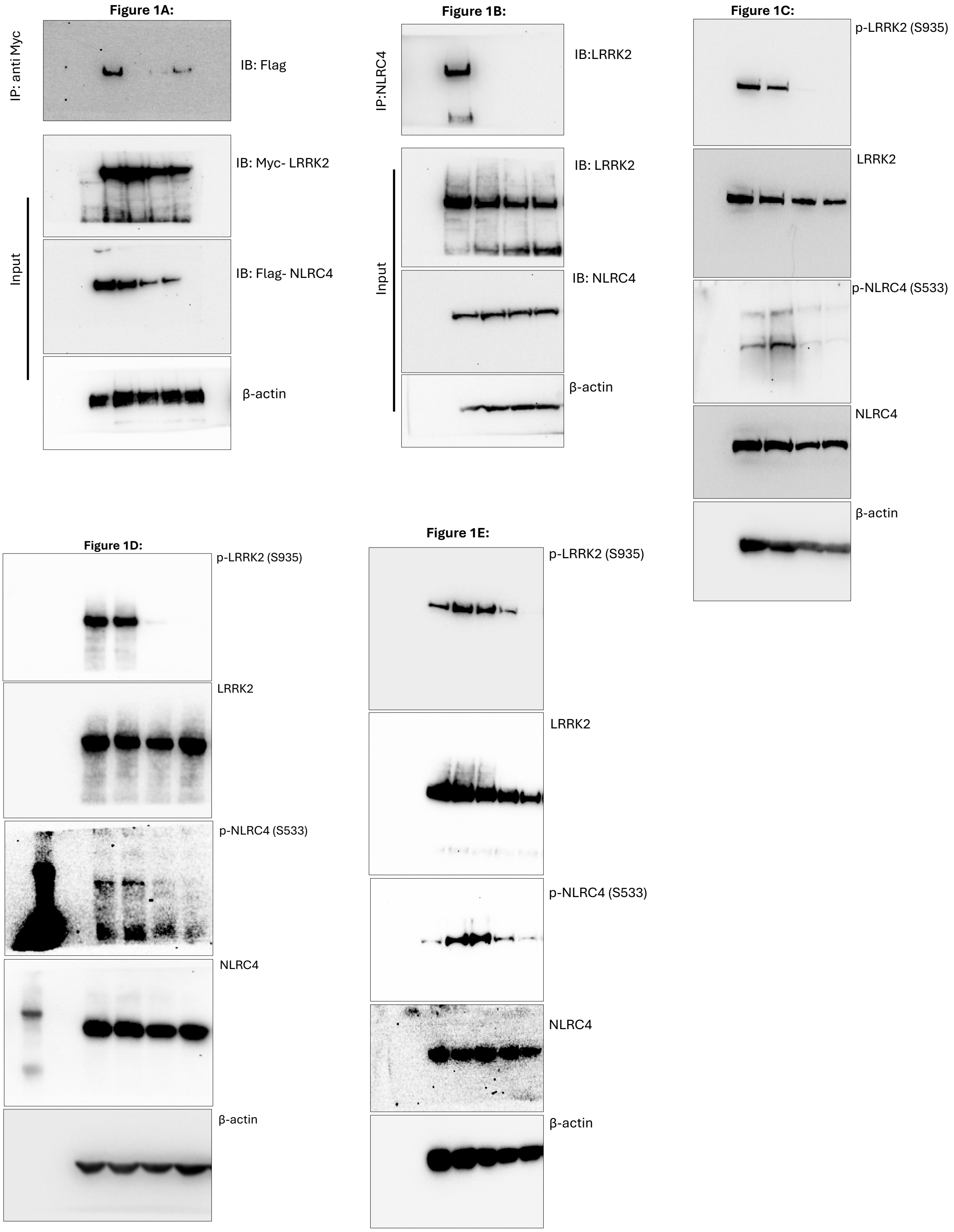

Supplement:

Figure 1F:

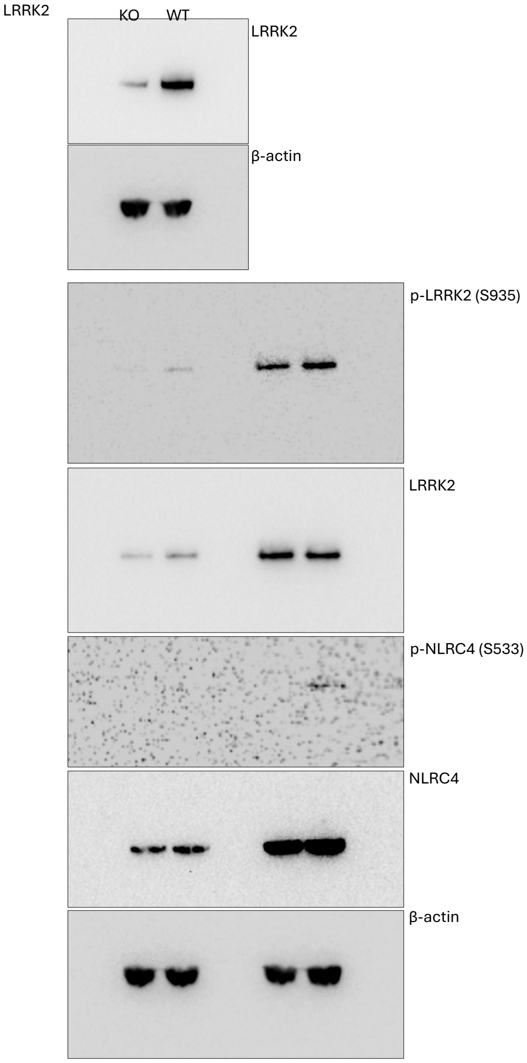

Figure 5A:

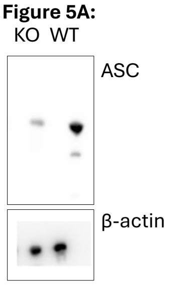

Figure 5B:

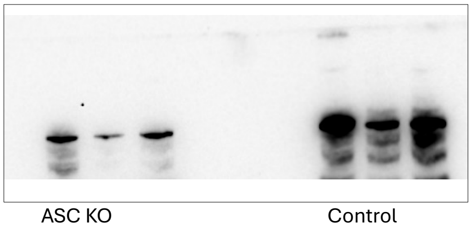

Supplement: Supplementary file 1 [file DataSheet1.pdf]
